# Targeting metabolic fluxes reverts metastatic transitions in ovarian cancer

**DOI:** 10.1016/j.isci.2023.108081

**Published:** 2023-09-28

**Authors:** Garhima Arora, Mallar Banerjee, Jimpi Langthasa, Ramray Bhat, Samrat Chatterjee

**Affiliations:** 1 Complex Analysis Group, Translational Health Science and Technology Institute, NCR Biotech Science Cluster, Faridabad 121001, India; 2 Developmental Biology and Genetics, Indian Institute of Science, Bangalore 560012, India; 3 BioSystems Science and Engineering, Indian Institute of Science, Bangalore 560012, India

**Keywords:** Physiology, Mathematical biosciences, Proteomics, Cancer

## Abstract

The formation of spheroids during epithelial ovarian cancer progression is correlated with peritoneal metastasis, disease recurrence, and poor prognosis. Although metastasis has been demonstrated to be driven by metabolic changes in transformed cells, mechanistic associations between metabolism and phenotypic transitions remain ill-explored. We performed quantitative proteomics to identify protein signatures associated with three distinct phenotypic morphologies (2D monolayers and two geometrically distinct three-dimensional spheroidal states) of the high-grade serous ovarian cancer line OVCAR-3. We obtained disease-driving phenotype-specific metabolic reaction modules and elucidated gene knockout strategies to reduce metabolic alterations that could drive phenotypic transitions. Exploring the DrugBank database, we identified and evaluated drugs that could impair such transitions and, hence, cancer progression. Finally, we experimentally validated our predictions by confirming the ability of one of our predicted drugs, the neuraminidase inhibitor oseltamivir, to inhibit spheroidogenesis in three ovarian cancer cell lines without any cytotoxic effects on untransformed stromal mesothelia.

## Highlights

• Proteomic signatures acquired for distinct morphological OVCAR-3 cell phenotypes
• Captured perturbed reaction modules associated with phenotype transition using GSMM
• Model predicts oseltamivir to reverse metabolic flux-driven phenotypic transition
• Oseltamivir inhibits spheroidogenesis across three ovarian cancer cell lines

## Graphical abstract

**Figure:**
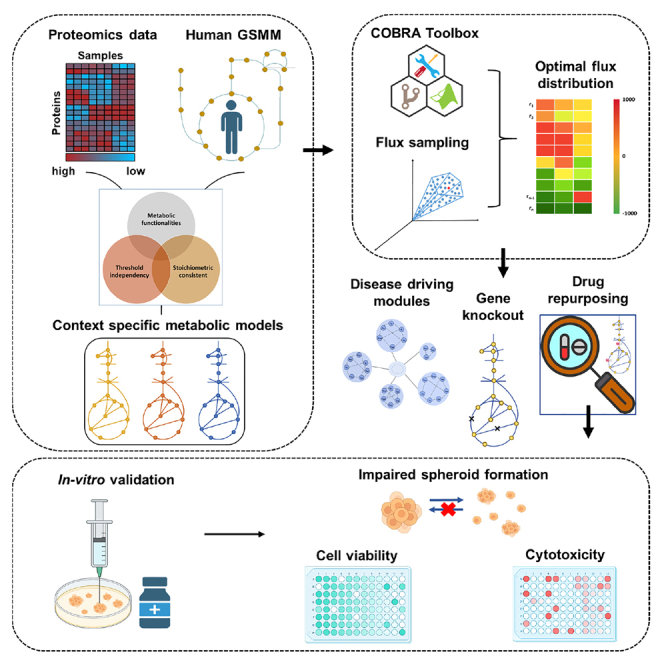

## Introduction

Epithelial ovarian cancer is one of the most common gynecological malignancies worldwide, with a high mortality rate and poor prognosis.^1^ Global estimates of ovarian cancer reveal an incidence of 300,000 new cases each year with an overall share of 1.6% of all cancers and an increased mortality rate.^1^^,^^2^ Delayed onset of clinical symptoms results in more than 70% of the cases left undiagnosed until cancer approaches an advanced stage with distant metastases. Auxiliary to this, the advanced stages of ovarian cancer culminate into a chemo-refractory form typically associated with poor overall survival.^3^ The metastasis of ovarian cancer begins with the detachment of transformed cells from the surface of the primary tumor due to decreased cell adhesion and accumulation within the peritoneal cavity.^4^ Changes in cell-cell adhesion follow a multi-step process of cancer progression, permitting epithelial cancer cells to migrate, remodel the extracellular matrix (ECM), and form clusters commonly known as spheroids. These spheroids contain cancer-associated fibroblasts and activated mesothelial cells, contributing to the development of the ascitic micro-environment.^5^ The existence of spheroids within the ascitic fluid highly correlates with the recurrence of the disease and resistance to the first-line platinum-based chemotherapeutic drugs.^6^ In fact, distinct spheroidal morphologies have been observed within patient ascites and using cell lines. Spheroids with a central cavitation are known as blastuloid spheroids, whereas those without any acellular center are called moruloid spheroids.^7^^,^^8^^,^^9^ The morphological distinction is due to the difference in ECM composition within the spheroids: moruloid spheroids show expression of fibronectin, whereas blastuloid spheroids have basement membrane matrix-like proteins. Morphological and molecular distinctions are associated with distinct behavioral phenotypes: whereas moruloid spheroids show liability and a greater tendency for matrix adhesion, blastuloid spheroids are resilient to stress.^10^

Alterations occurring in tumor cell mechanics require metabolic rewiring along this process to satisfy cancer cell’s energetic needs, which is one of the hallmarks of epithelial cancers. ^13^C metabolic studies showed that spheroid forming cells increase anaerobic glycolysis as well as pentose cycle and decrease re-routing of glucose for anabolic purposes.^11^ This allows spheroidal cells to proliferate more aggressively and show an enhanced resistance to apoptosis. The amino acids, namely, glutamine, glutamate, serine, and aspartate, which are essential for carrying out tricarboxylic acid (TCA) cycle reactions, were significantly increased in spheroid-forming ovarian cancer cells compared to cancer cells cultured in adherent plates.^12^ Taken together, distinct signatures of metabolite levels in cancer spheroids suggest that specific flux dynamics of metabolites may be associated with spheroid formation, and hence, spheroidogenesis can be disrupted by targeting such fluxes.

Studies concerning metabolic alterations have used metabolic models to investigate different metabolic states of cells under normal and cancerous conditions and advance our ability to identify potential drug targets and biomarkers.^13^^,^^14^^,^^15^ Pan-cancer analysis done by Gatto et al.,^16^ involving 13 different cancers, including ovarian, shows the general metabolic features separating normal and cancerous phenotypes. The study builds an insightful metabolism-driven narrative for cancer progression but does not explore tissue-specific cancer manipulations. Motamedian et al. have shown significant metabolic differences in the metabolism of cisplatin-sensitive and -resistant epithelial ovarian cancer cells and normal ovarian epithelia using a genome-scale metabolic model (GSMM).^17^ Evidently, few studies have been done on genome-scale metabolic models in ovarian cancer. In addition, to the best of our knowledge, there are no studies examining stage- or morphology-specific changes in metabolic dynamics of ovarian cancer.

In order to identify metabolic perturbations during the phenotypic transitions, we performed quantitative proteomics to delineate distinct protein signatures between monolayer cultures of an ovarian cancer cell line OVCAR-3 and its two distinct spheroidal morphologies (see Data S1). Our objective was to predict the transition-specific perturbation modules responsible for disease progression and disrupt it through targeting its regulatory points. We reconstructed context-specific metabolic models for OVCAR-3 samples of three morphological stages using Recon3D^18^ and examined the metabolic modulation associated with disease progression. We then explored the knockout strategies in these metabolic networks to reduce tumor-specific metabolic alterations during ovarian cancer progression. We investigated the DrugBank database^19^ to extract drugs against enzymes catalyzing the metabolic reactions and evaluated their effect in impairing ovarian cancer progression using the metabolic models of each spheroidal morphology. Finally, we experimentally validated our model predictions on the pharmacological regulation of metastatic spheroidal morphologies from multiple ovarian cancer cell lines.

## Results

### OVCAR-3 proteomics data reveal perturbations associated with morphological transitions

**Figure 1:**
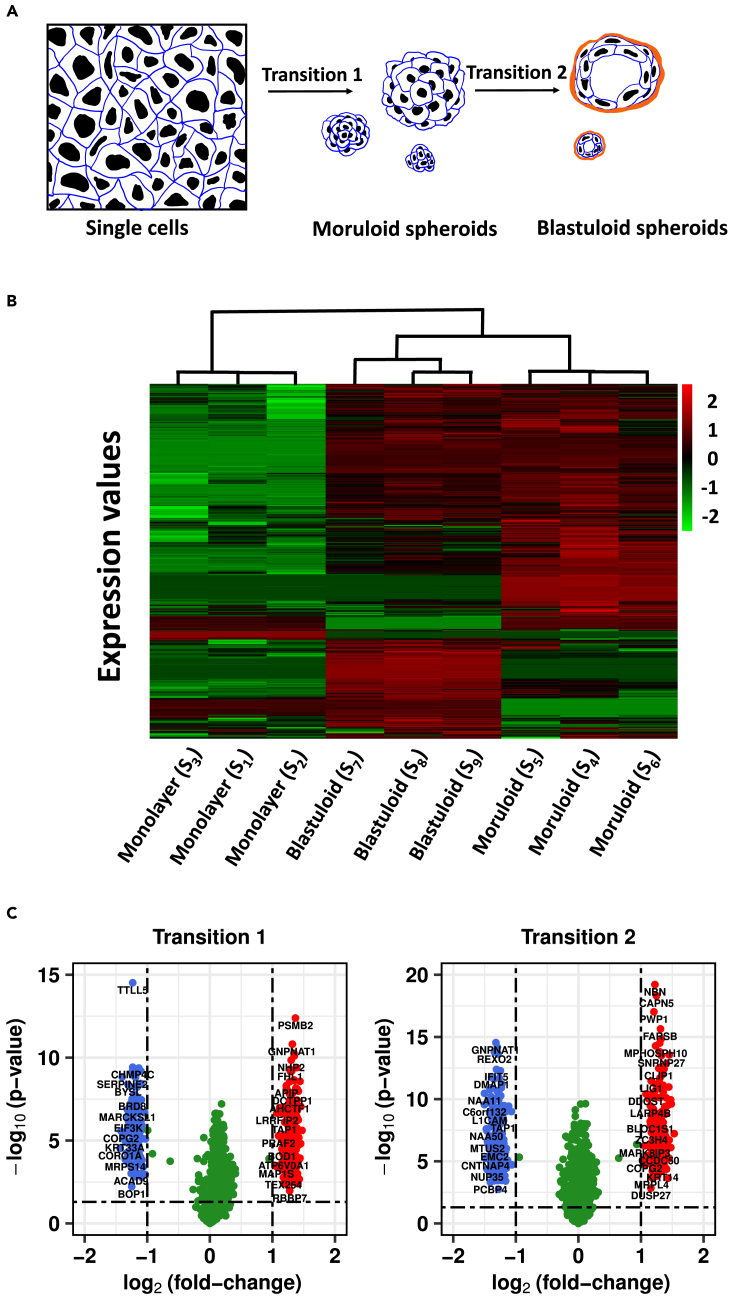
Proteomic changes during morphological transitions in OVCAR-3 ovarian cancer cells (A) Cartoon depiction of the single cell-to-moruloid and moruloid-to-blastuloid transitions. (B) Heatmap showing standardized protein expressions across all 9 (3x3) samples. The color bar shows the standardized protein expression value, and the dendrogram represents hierarchical clustering among the samples of morphological stages of OVCAR-3. (C) Volcano plots showing the distribution of differentially expressed proteins for both the morphological stage transitions. The X axis represents $\log_{2}$ (fold-change) of expression values between the profiles, and the Y axis represents ${-\log}_{10}$ (p value) obtained from a t-test of differences between the samples. The horizontal dashed line represents p value = 0.05, whereas vertical dashed lines partition the plot for |$\log_{2}$ (fold-change) |≥1. Blue and red dots represent the down-regulated and the up-regulated proteins, respectively, whereas green dots represent not differentially expressed proteins.

High-throughput proteomics was performed in this study using the mass spectrometry data-dependent top10 approach. The data contains the expression of 3,122 proteins across the three biological replicate samples of each morphological stage of the OVCAR-3 cell line (see Figure 1A), namely, monolayer, moruloid (24-h spheroids), and blastuloid (1-week spheroids) (see STAR methods section). The proteomics data relating to both the monolayer-moruloid transition and the moruloid-blastuloid transition is provided in Data S1. A part of this proteomics data related to moruloid-blastuloid transition is also provided in a previous study.^7^ The proteomics data was first pre-processed in order to account for missing data and variability (see STAR methods section). The protein expressions showed varying perturbation levels during the initial and later stages of the morphological transition of the OVCAR-3 cell line. Agglomerative hierarchical clustering revealed grouping among the replicates of each morphological stage, trailed by clustering in samples of moruloid and blastuloid spheroids (see Figure 1B).

Differentially expressed proteins between progressive morphological stages of OVCAR-3 were identified by applying a cutoff of 1 on $\log_{2}$ fold-change of protein expression values (see Figure 1C). Among the top perturbed proteins, GNPNAT1, CTBP1, GMPR, and SLC25A6 were found to be commonly perturbed during both transitions. During transition 1, GLS, DBI, and STK4 were among the top perturbed enzymatic proteins, whereas, during transition 2, enzymatic proteins namely, ILK, MAP2K1, and AHCYL1 were perturbed. Using Enrichr,^20^ pathway enrichment analysis of differentially expressed proteins was done for both transitions (see Data S2). Metabolic reprogramming is the major hallmark of cancer; however, its role in different morphological stages of OVCAR-3 might help us to understand the tumor progression in ovarian cancer.

### Generation of context-specific metabolic models for each morphological stage

To acquire a better understanding of how the morphological changes during tumor progression are associated with metabolic reprogramming, we used a generic human metabolic model Recon3D.^18^ Choosing an efficient model extraction method (MEM) to create context-specific models has always been challenging because it considerably impacts on the size and functionality of the context-specific models.^21^ Cells perform distinct metabolic functions under different pysiological conditions; hence pre-defining a specific metabolic objective of a cell for all conditions is not appropriate. Moreover, previous experimental observation also suggested that during the transition from moruloid spheroids to blastuloid spheroids, the phenotypes did not exhibit any significant difference in cell proliferation.^7^ Therefore, to build metabolic models for each morphological stage of the OVCAR-3 cell line, we have used three different MEMs, namely iMAT,^22^ FASTCORE,^23^ and INIT,^24^ that are independent of the objective function and each with two different thresholds (see STAR methods section). The number of reactions and metabolites present in all the metabolic models and the threshold values used to extract models for each morphological stage are given in Data S3.

**Figure 2:**
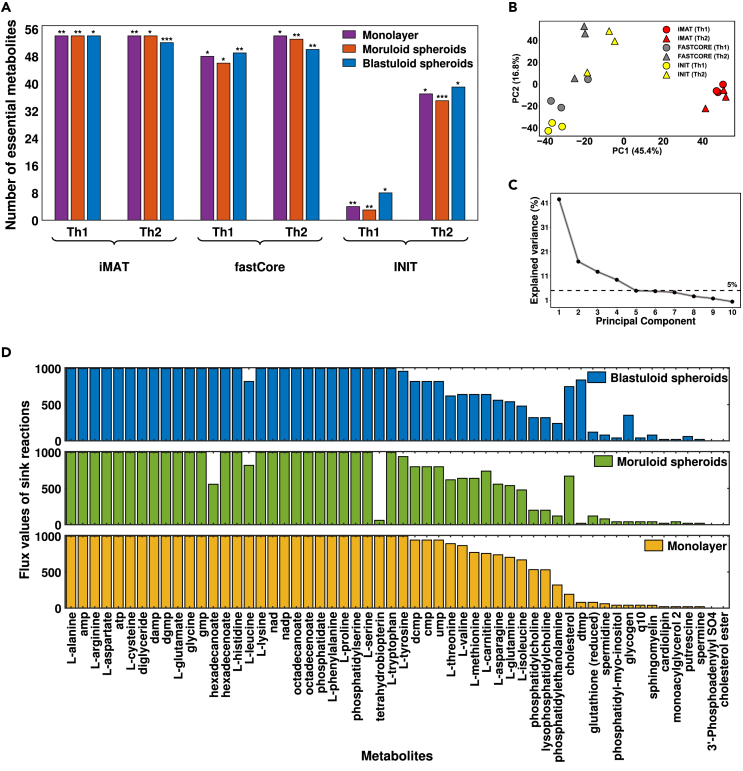
Systematic evaluation of different Model Extraction Methods (MEMs) (A) The 56 metabolites essential for cancer growth^21^ encoded as a biomass function were taken as a surrogate for every possible metabolic function. The bar graph shows significant metabolic functionality scores, i.e., the number of essential metabolic functions for cancer growth having non-zero flux values corresponding to each model. Here, Th1: Threshold-1, Th2: Threshold-2. The significance of the metabolic functionality score (see STAR methods section) for each model is marked at the top of each bar (∗p < 0.05, ∗∗p < 0.01, ∗∗∗p < 0.001). (B) PCA plot represents the clustering of metabolic models for different morphological stages of the OVCAR-3 cell line based on the MEMs and threshold values. The red dots in the PCA plot represent models built using the iMAT technique, whereas the gray and yellow dots represent models built using FASTCORE and INIT, respectively. (C) The line plot depicts the percentage variance explained in each principal component. (D) Bar graphs showing flux profiles of sink reaction corresponding to each metabolite essential for cancer growth in iMAT-Th1 models of each morphological stage.

The central premise of these MEMs is to extract reactions corresponding to highly expressed genes preferentially. However, cellular metabolic functionalities are not always preserved after model extraction. The study by Opdam et al.^21^ provided 56 metabolites based on the biomass function that are essential for cancer cell growth. These metabolites include the synthesis of non-secreted metabolites, e.g., glutathione, ATP, and carnitine. Following this study, we evaluated the metabolic functionality score (see STAR methods section) for all the afore-built context-specific models (see Figure 2A). Furthermore, a multivariate PCA analysis of the steady state reaction’s flux values in all the models revealed their dependencies on the choice of MEM and threshold (see Figures 2B and 2C). It was observed that the models built using the iMAT method had a high metabolic functionality score and were less sensitive to threshold selection. For all three morphological stages of the OVCAR-3 cell line, iMAT models with threshold 1 (iMAT-Th1) were chosen to gain further insights into the metabolic liabilities. The flux values of sink reactions corresponding to 56 metabolites based on biomass function in all three morphological stage-specific iMAT-Th1 models are illustrated in Figure 2D. During the phenotypic transition from monolayer to spheroidal morphologies, the flux values of the sink reaction associated with the metabolite cholesterol increased significantly. On the other hand, the flux values of the sink reaction corresponding to asparagine, threonine, leucine, and valine decreased.

### Metabolic profiling of the iMAT-Th1 models translates morphological transitions into metabolic perturbations

**Figure 3:**
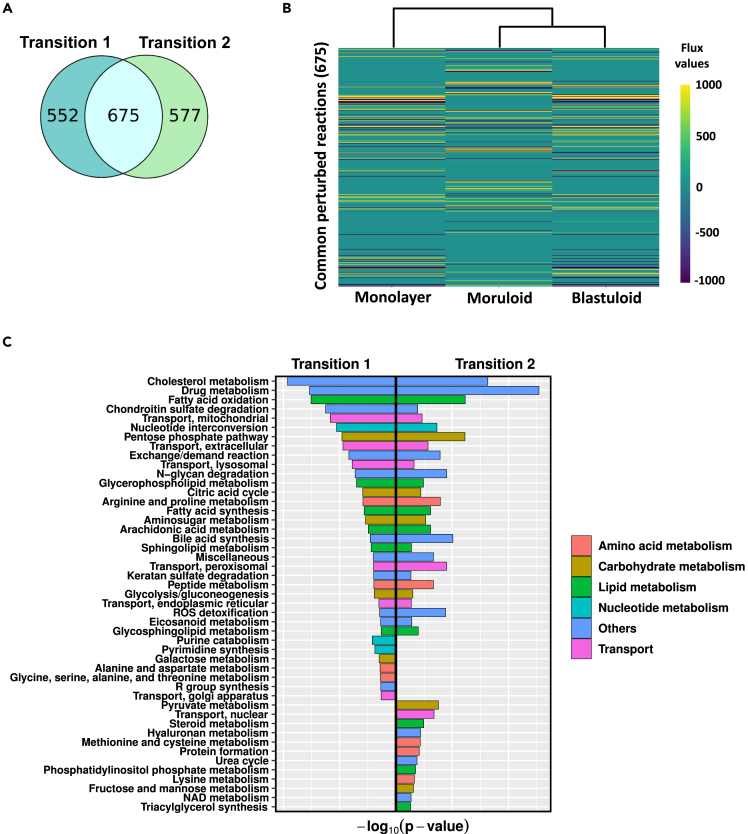
Metabolic perturbations in steady-state flux profile of iMAT-Th1 models (A) Venn diagram shows the number of common and specific reactions perturbed during each transition. (B) Heatmap represents the steady-state flux values of the reactions commonly perturbed (675) during both the transitions and the hierarchical clustering among the metabolic profiles of the morphological stages. (C) The figure shows the pathways significantly perturbed (p value < 0.05) during both transitions. The color coding represents the presence of pathways involved in different metabolic processes.

Following the results from the previous section, the iMAT-Th1 models of all three morphological stages were considered for further investigation. The iMAT-Th1 models had a high metabolic functionality score and were less sensitive to the threshold selection; therefore, they were considered for further investigation. Constraint-based modeling (CBM) flux sampling technique was used to evaluate the steady-state flux values,^25^ and the set of flux values with the highest correlation with the input expression data were considered as the optimal solution (see STAR methods section). A two-fold cut-off was applied to flux fold-change values of reactions present in the models of both stages to determine reactions perturbed during each transition. It was found that 1226 and 1252 reactions were perturbed in transitions 1 and 2, respectively (see Figure 3A). The flux value of 675 reactions commonly perturbed during both morphological stage transitions has been represented through the heatmap (see Figure 3B). The clustering among moruloid and blastuloid profiles was retained after integrating proteomics data into the metabolic model (see Figure 3B). The metabolic pathway enrichment analysis of perturbed reactions was done using the Recon3D database. The significance of overlap was evaluated using the hypergeometric test. The enriched pathways were further categorized based on their participation in metabolic processes. Lipid, carbohydrate, and protein/amino acid metabolism contributed a significant share in the progression of the disease (see Figure 3C). Several pathways were impaired during both transitions, including glycolysis, TCA, fatty acid synthesis and oxidation, cholesterol metabolism, and a few transport mechanisms. Pyrimidine synthesis and purine catabolism were only disrupted during transition 1 among nucleotide metabolism-associated pathways. Pathways involved in amino acid metabolism, such as alanine & aspartate and glycine, serine, alanine, and threonine metabolism, were significantly perturbed, specifically in transition 1. On the other hand, for transition 2, specific perturbation in methionine & cysteine, lysine metabolism was observed. During transition 2, alterations in pyruvate metabolism, a part of carbohydrate metabolism, were also discovered. Moreover, a few pathways involved in lipid metabolism, namely, triacylglycerol synthesis and steroid metabolism, were also significantly altered during transition 2.

### Identification of core reaction modules associated with disease progression

**Figure 4:**
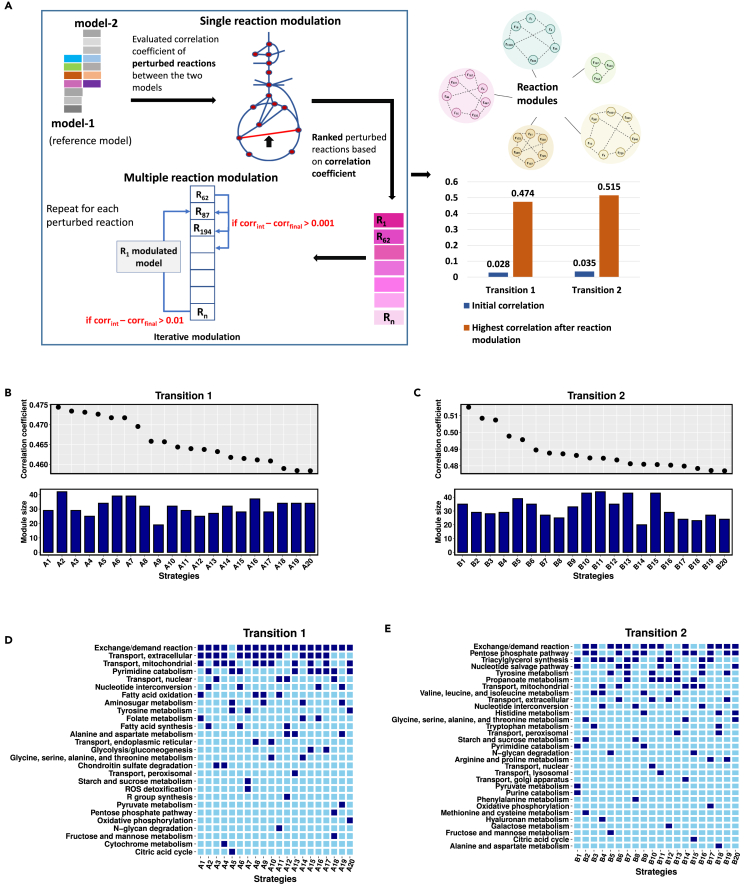
Grouping metabolic perturbed reactions based on their influence on morphological transitions (A) Schematic diagram summarizes the reaction modulation process followed during the search for perturbed reaction modules responsible for the disease transition. (B and C) The figures show the correlation coefficient obtained corresponding to the top 20 reaction modulation strategies and the number of reactions modulated in each strategy. (D and E) The plot shows the pathways involved in the top 20 modulation strategies (Ai and Bi, i = 1,2,..,20) for both transitions. The involvement of a pathway in a particular strategy is represented by dark blue color, whereas light blue color represents its absence.

Metabolic alterations drive OVCAR-3 cancer cells to gain a metastatic phenotype and form spheroids in the peritoneal cavity. Perturbations directing the transition of morphological stages of the OVCAR-3 cell line were obtained in previous section. A low correlation of 0.0284 and 0.0352 ascertained the significant shift in the metabolic flux profiles during transitions 1 and 2, respectively. Both common and transition-specific reactions were large in number, and targeting such large perturbations is biologically infeasible. Therefore, we defined an approach for finding an optimal set of metabolic reactions whose perturbation is responsible for the transition of the morphological stages of the OVCAR-3 cell line. This technique was aimed at finding small modules of reactions whose modulation increases the correlation between the flux profiles of perturbed reactions in the modulated and initial stage model of the transition (see Figure 4A). In single reaction modulation, the mean correlation between the perturbed flux profiles during transition 1 and transition 2 were 0.0767 and 0.0896, respectively, which is not a very significant increase. This reiterated the fact that the disease alters the cellular metabolism at multiple sites; hence, simultaneous modulation of multiple reactions should be an optimal approach. So, we performed multiple reaction modulation (see STAR methods section), and the maximum correlations achieved for both transitions were 0.4744 and 0.5151, respectively. The number of modulated reactions and the associated correlation coefficient obtained after modulation were found to have no relationship (see Figures 4B and 4C). This means that to achieve high correlation, it is not necessary to modulate a large number of reactions. Hence, the impact of each strategy is not dependent on how many reactions are modulated; rather, it depends on the reaction combination.

The reactions present in top modulation strategies (see Data S4) of both the transitions were involved in the exchange/demand reaction pathway, and a few transport pathways such as transport extracellular, transport mitochondrial (see Figures 4D and 4E). Carbohydrate related pathways such as citric acid cycle (TCA), pentose phosphate, oxidative phosphorylation, pyruvate metabolism, and pathways involved in amino acid metabolism such as tyrosine, alanine & aspartate metabolism were involved in top modulation strategies of both the transitions. In addition, when comparing transition 2 to transition 1, reactions involved in the pentose phosphate pathway were found in many of the top modulation strategies. Only transition 1’s top modulation strategies showed evidence of glycolysis/gluconeogenesis, fatty acid oxidation, and synthesis. The involvement of the triacylglycerol synthesis pathway (TAG) was observed in 10 of the top 20 modulation strategies for transition 2. Arginine and proline pathway involved in amino acid metabolism was also involved in transition 2’s top modulation strategies.

### Predicting drug targets in reversing the metabolic alterations during OVCAR-3 morphological transitions

**Figure 5:**
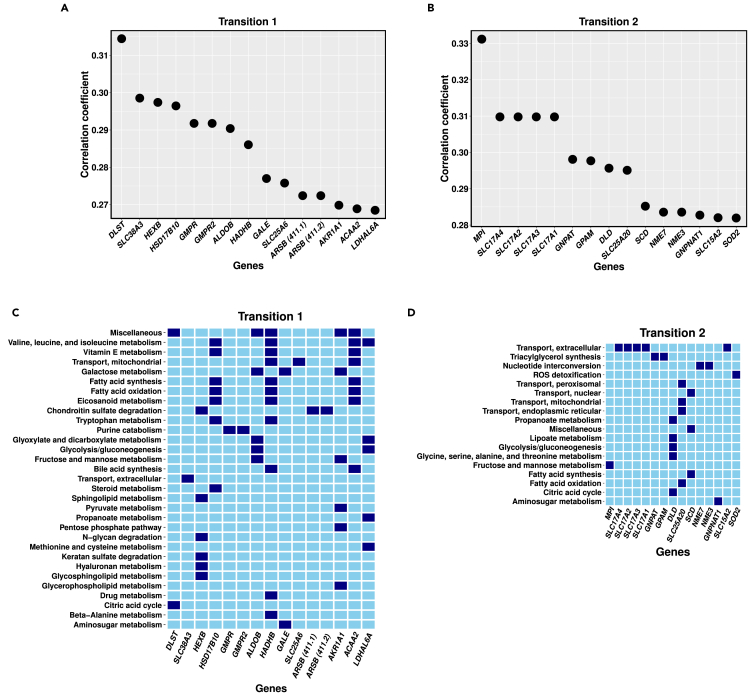
Identification and *in silico* knockout of targets identified through correlation analysis and their catalyzed pathways (A and B) The figures show the top 15 gene knockout targets based on the correlation coefficient. (C and D) Pathways enriched by reactions catalyzed by the top 15 gene knockout targets are depicted in the figure. The gene’s involvement in the corresponding pathway is represented by dark blue, whereas light blue color represents its absence.

Alterations in the flux values of metabolic reactions were observed during the transition from the initial stage to the later stages of OVCAR-3. Targeting genes catalyzing the perturbed reactions can be a potential strategy for reversing the metabolic alterations that occur during morphological transitions. For this, we filtered out a total of 651 and 626 genes catalyzing reactions that were perturbed during transition 1 and transition 2, respectively. We sifted 495 and 462 non-essential genes (see STAR methods section for details) to perform single gene knockouts in the iMAT-Th1 models of moruloid and blastuloid spheroids, respectively. The metabolic flux profiles after single gene knockout were then examined and compared to the flux profiles of the transition’s initial stage model. After executing single gene knockout using genes catalyzing reactions altered during transitions 1 and 2, the highest correlation coefficients obtained were 0.31447 and 0.33119, respectively (see Figures 5A and 5B).

Silencing of genes involved in carbohydrate metabolism pathways, namely, *DLST*, *GALE* showed a significantly high correlation, i.e., were able to rectify the metabolic profile altered during transition 1. Multi-pathway targeting genes *HSD17B10*, *HADHB*, and *ACAA2*, were among the top 5 gene knockout results of transition 1 (see Figure 5C). Silencing of many solute carriers (*SLC*) genes such as *SLC17A4*, *SLC17A2*, *SLC25A1*, and others (see Data S5) that encodes membrane transport proteins, were able to reduce the metabolic perturbations occurred during transition 2. Interestingly, knocking down of genes *GNPAT* and *GPAM* implicated in the triacylglycerol synthesis pathway (lipid metabolism) were among the top gene knockout results of transition 2 (see Figure 5D). This follows our finding in the previous section that the triacylglycerol pathway undergoes a significant amount of perturbation, as its reactions were prominent among the top reaction modulation strategies of transition 2. We also observed that a single gene knockout of *ALDOB*, *ADH1B*, *NEU1*, and *NT5E* minimized the metabolic changes during both transitions.

Proteins essential to cancer survivability are of great interest in the search for new therapeutic targets.^26^^,^^27^ To investigate the effect of targeting such essential protein-encoding genes in ovarian cancer, we retrieved 36 metabolic genes from the study by Kanhaiya et al.^28^ These genes were subjected to single gene knockout in our iMAT-Th1 models of moruloid and blastuloid spheroids of OVCAR-3. We observed an increase in correlation between the flux profiles of perturbed reactions in the acquired model following gene knockout and the initial model of the transition, i.e., $\log_{2}$ fold-change in correlation was greater than 2. Ensuing gene essentiality results, we assigned a targetability score to the top 20 reaction modulation strategies of both transitions. The percentage of the non-essential genes involved in each modulation strategy was defined as the targetability score (see Tables S1 and S2). Additionally, the pathways enriched by reactions in the top 20 reaction modules, catalyzed by non-essential genes, are provided in Figures S1A and S1B.

### Investigating the effect of drugs in reversing the disease progression

**Figure 6:**
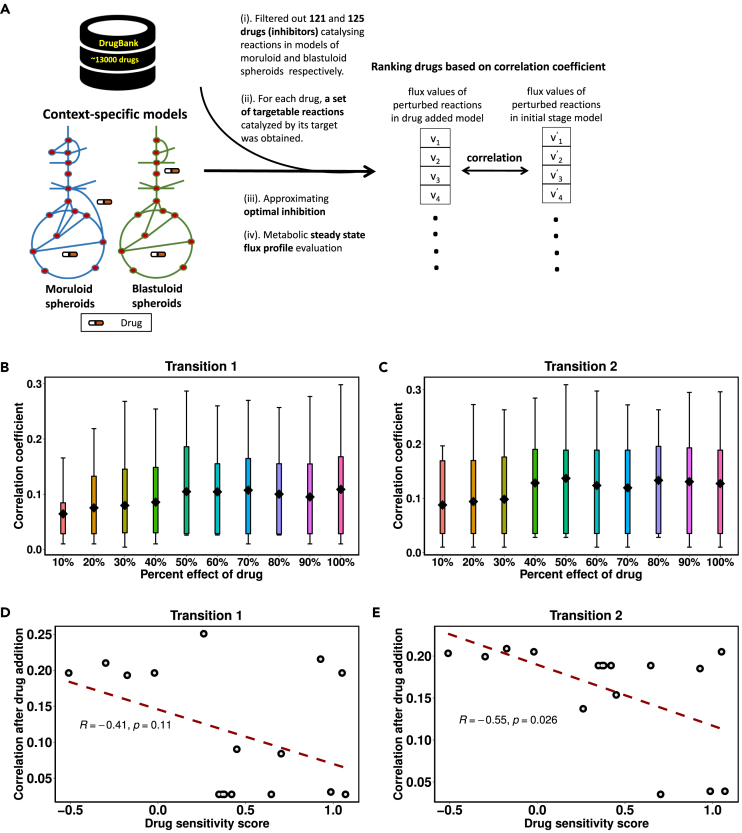
Estimation of the effect of repurposable drugs in altering the metabolic state of ovarian cancer cells using the metabolic models (A) The workflow for evaluating the effect of drug response in the metabolic models. (B and C) The figure shows the percentage effect of the drug over the model readouts after adding the drug. (D and E) The regression plot shows an inverse relationship between the model readout, i.e., the correlation coefficient corresponding to 16 drugs and the drug’s sensitivity score obtained from GDSC. A positive score implies low sensitivity of the OVCAR-3 cell line, whereas a negative score implies a high sensitivity of the OVCAR-3 cell line toward the drug administration.

Drug repurposing, also known as re-profiling, is instrumental in exploring new therapeutic uses for approved or investigatory drugs for a new medical indication. To perform in-silico drug repurposing in the models of moruloid and blastuloid spheroids, we extracted the information of 13,000 drugs and their corresponding targets from the DrugBank database.^19^ We were able to map targets of 121 and 125 drugs (inhibitors) to the metabolic reactions catalyzed by them, respectively, in models of moruloid and blastuloid spheroids (see Figure 6A). The effect of a drug (inhibitor) in the models was evaluated by reducing the flux bounds of reactions catalyzed by the metabolic targets of the drug. It was observed that the mean correlation coefficient obtained from the drug infused models increased up to 50% inhibition, and a further increase in percentage inhibition has little or no effect on the flux profiles (see Figures 6B and 6C). Therefore, 50% reaction bound inhibition was chosen to achieve optimum efficacy during drug repurposing (see STAR methods section). For each drug, the correlation coefficient between the flux values of perturbed reactions in drug infused model and the initial stage model of a transition was evaluated (see Data S6).

We filtered out drugs having targets overlapped with the gene list utilized for gene knockout and having log_2_ fold-change in correlation greater than 2 (see Tables 1 and 2). Among the drug repurposing results, single gene targeting drugs, namely disulfiram, ethoxzolamide, gadopentetate dimeglumine, and tolcapone, were specific to transition 1, whereas amphetamine, cefdinir, and cerulenin were specific to transition 2. The single gene targeting drugs canagliflozin, moexipril, oseltamivir, myo-inositol, glyburide, gemcitabine, pentoxifylline, and salicylic acid target the genes involved in metabolic alterations occurring in both the disease transitions. Few multi-target drugs, such as benzthiazide, clodronate, fomepizole, hexachlorophene, methyclothiazide, and troglitazone, with all their targets involved in the reactions, perturbed during transition 1. Clodronate and fomepizole, on the other hand, were the only two multi-target drugs, having all their targets present in the metabolic perturbation developed during transition 2.

**Table 1:** Drug candidates targeting metabolic perturbations during transition 1

Drugs	Target (Gene ID)	Target (Gene Name)
Glyburide	19.1	*ABCA1*
Gemcitabine	51727.1	*CMPK1*
Canagliflozin	6523.1	*SLC5A1*
Disulfiram	217.1	*ALDH2*
Tolcapone	1312.1	*COMT*
Moexipril	59272.1	*ACE2*
Ethoxzolamide	766.1	*CA7*
Gadopentetate dimeglumine	5226.1	*PGD*
Oseltamivir	4758.1	*NEU1*
Myo-Inositol	10423.1	*CDIPT*
Pentoxifylline	4907.1	*NT5E*
Salicylic acid	1645.1	*AKR1C1*
Benzthiazide	771.1, 768.1	*CA12, CA9*
Clodronate	291.1, 292.1, 293.1	*SLC25A4, SLC25A5, SLC25A6*
Fomepizole	124.1, 125.1, 126.1, 847.1	*ADH1A, ADH1B, ADH1C, CAT*
Hexachlorophene	2746.1, 6392.1	*GLUD1, SDHD*
Methyclothiazide	759.1, 760.1, 762.1	*CA1, CA2, CA4*
Troglitazone	2182.1, 2030.1	*ACSL4, SLC29A1*

**Table 2:** Drug candidates targeting metabolic perturbations during transition 2

Drugs	Target (Gene ID)	Target (Gene Name)
Canagliflozin	6523.1	*SLC5A1*
Cerulenin	2194.1	*FASN*
Cefdinir	4353.1	*MPO*
Moexipril	59272.1	*ACE2*
Oseltamivir	4758.1	*NEU1*
Myo-Inositol	10423.1	*CDIPT*
Amphetamine	4128.1	*MAOA*
Glyburide	19.1	*ABCA1*
Gemcitabine	51727.1	*CMPK1*
Pentoxifylline	4907.1	*NT5E*
Salicylic acid	1645.1	*AKR1C1*
Clodronate	291.1, 292.1, 293.1	*SLC25A4, SLC25A5, SLC25A6*
Fomepizole	124.1, 125.1, 126.1, 847.1	*ADH1A, ADH1B, ADH1C, CAT*

We compared our model’s predicted drug response with the drug sensitivity scores (IC50 *Z* score) obtained from Genomics of Drug Sensitivity in Cancer (GDSC) for the OVCAR-3 cell line.^29^ The IC50 *Z* score describes whether a cell line is sensitive or resistant to a drug compared to other cell lines screened against the same drug. A negative score means the cell line is more sensitive to the drug, while a positive score means less sensitive. The *Z* score of a drug for a particular cell line is defined as:

$$z=\frac{x-\mu }{\sigma^{2}}$$

where, *x* is the IC50 value of the drug in question (over OVCAR-3 cell line), μ is the mean and $\sigma^{2}$ is the standard deviation of the IC50 values for the drug in question over all cell lines treated. From our drug list, we were able to obtain the drug sensitivity scores (IC50 *Z* score) for only 16 drugs (given in Tables S3 and S4). The correlation coefficient (obtained after adding the drug to the model) for these 16 drugs showed an inverse relationship with their corresponding z-scores from the GDSC database. This implies that drugs with negative *Z* score in the GDSC database had higher correlation through our model, suggesting that the OVCAR-3 cell line is more sensitive toward the drugs which have a high correlation coefficient (see Figures 6D and 6E). These results showed that the model predicted drug response also asserts the responsiveness of a drug toward the OVCAR-3 cell line. Single-target drugs common to both transitions were chosen (from Tables 1 and 2), and pathway enrichment analysis was carried out to gain mechanistic insights using the reactions catalyzed by the targets of these drugs. It was observed that the drug oseltamivir targeting neuraminidase was involved in the highest number of pathways as compared to other drugs (see Data S7). We also evaluated the overlap value for each pathway, which is calculated as the ratio of the number of reactions targeted by the drug in that pathway to the total number of reactions in that pathway. Oseltamivir showed larger overlap values and higher pathway perturbation than the other drugs.

### Experimental validation of drug repurposing predictions

**Figure 7:**
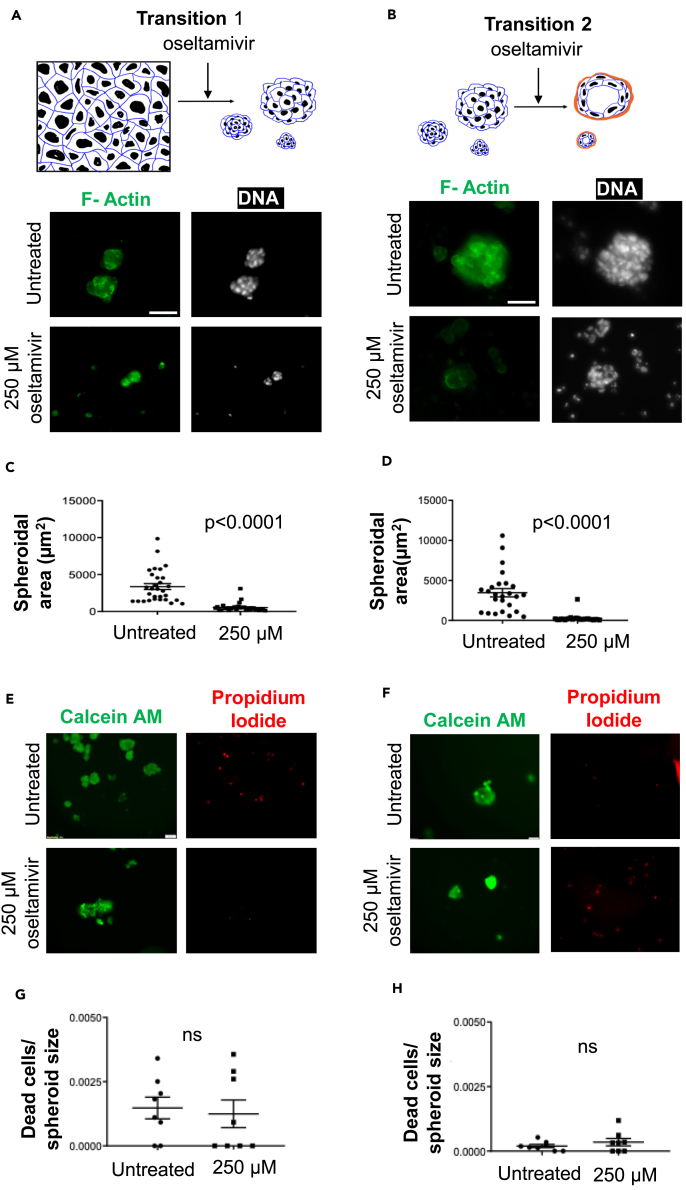
Oseltamivir treatment disrupts single cell-to-moruloid and moruloid to-blastuloid transitions of ovarian cancer spheroids (A) Confocal photomicrographs of ovarian cancer OVCAR-3 cells allowed to transit from single cells to moruloid spheroids (transition 1; top represents a cartoon representation of the same). Cells were untreated (top row of micrographs) or treated with 250 μM oseltamivir (second row from top) and observed after 48 h of culture with staining for F-actin (green using phalloidin) and DNA (white using DAPI) (n = 3). (B) Confocal photomicrographs of ovarian cancer OVCAR-3 cells allowed to transit from moruloid to blastuloid spheroids (transition 2; top represents a cartoon representation of the same). Spheroids were untreated (top row of micrographs) or treated with 250 μM oseltamivir (second row from top) and observed after 48 h of culture with staining for F-actin (green using phalloidin) and DNA (white using DAPI) (n = 3). Scale bar: 50 μm. (C) Graph showing the spheroidal size of 250 μM oseltamivir treated cells compared untreated cells during transition 1 (n = 3, N = 30 spheroids). (D) Graph showing the spheroidal size of 250 μM oseltamivir treated cells compared untreated cells during transition 2 (n = 3, N = 30 spheroids). (E) Epifluorescence photomicrographs of ovarian cancer OVCAR-3 cells untreated (top row of micrographs) or treated with 250 μM oseltamivir (second row from top) and observed after 48 h of culture with staining by Calcein AM (green; marker for viability) and propidium iodide (red; marker for cell death) (n = 3). (F) Epifluorescence photomicrographs of ovarian cancer OVCAR-3 cells untreated (top row of micrographs) or treated with 250 μM oseltamivir (second from top) and observed after 48 h of culture with staining by Calcein AM (green; marker for viability) and propidium iodide (red; marker for cell death) (n = 3). Scale bar: 50 μm. (G) Graph numbers of dead cells within a spheroid normalized to its size between untreated and 250 μM oseltamivir treated cells during transition 1 (n = 3, N = 8 spheroids). (H) Graph showing numbers of dead cells within a spheroid normalized to its size between untreated and 250 μM oseltamivir treated cells during transition 2 (n = 3, N = 8 spheroids). Error bars denote mean ± SEM. The unpaired Student’s *t* test with Welch’s correction was performed for statistical significance.

**Figure 8:**
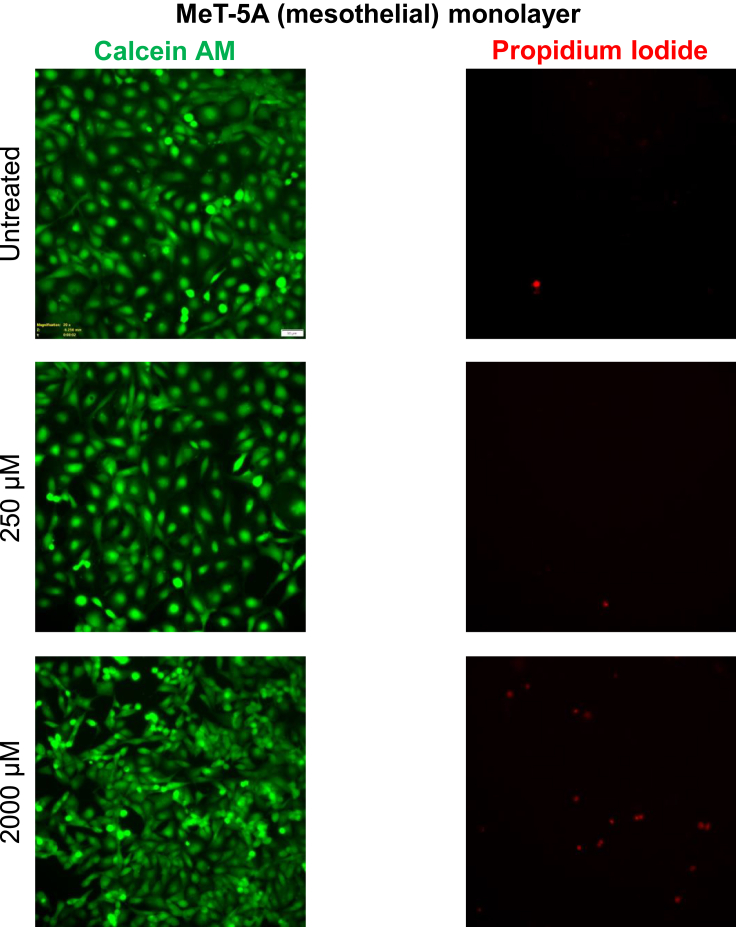
Spheroid disrupting doses of oseltamivir are not cytotoxic to mesothelial MeT-5A monolayers Epifluorescence photomicrographs of the untransformed mesothelial line MeT-5A cultivated as monolayers: untreated (top row), treated with 250 μM oseltamivir (middle row), and with 2000 μM oseltamivir (bottom row) and observed after 48 h of culture with staining by Calcein AM (green; marker for viability) and propidium iodide (red; marker for cell death) (n = 3). See Table S5 for quantification of cytotoxicity due to drug exposure.

To validate our prediction on the pharmacological regulation of both transition 1 and transition 2, we chose oseltamivir, which was associated with both transitions with large overlap values and high pathway perturbation. It targets neuraminidase which has been demonstrated to contribute to the progression of various cancers^30^^,^^31^^,^^32^ in a context-dependent manner (see also^33^) for a progression inhibitive role of oseltamivir. Although the levels of Neuraminidase-1 have been correlated with ovarian cancer cell proliferation and invasion,^34^ an association between the ability of its enzymatic activity to regulate the sialic acid flux and the formation of spheroids, the principal mediators of ovarian cancer metastasis,^35^ as revealed through the global metabolic analysis of the spheroidal proteomics is as yet unknown. Therefore, we chose this drug for further *in vitro* evaluation in ovarian cancer. Using a resazurin assay the IC50 for oseltamivir treatment of OVCAR-3 was ascertained to be 1.7 mM (see Figure S2A). Progressively increasing concentrations of oseltamivir were added to suspended single OVCAR-3 cells, which were cultured for 48-h. Untreated control cells formed moruloid spheroids within this time period (Figure 7A). On the other hand, moruloid spheroid formation (transition 1) was impaired in oseltamivir-added cultures upon treatment with sub IC50 concentrations of 250 μM (morphology and cellularity assessed using staining for F-actin using phalloidin and for DNA using DAPI, graph showing the change in spheroid size shown in Figure 7C; 2 mM EGTA was used as a positive control as it disrupts spheroid formation see Figure S2B). Upon treatment of already-formed moruloid spheroids with progressively increasing concentrations of oseltamivir, disruption of transition 2 was observed with sub IC50 concentrations 250 μM (Figure 7B) (morphology and cellularity assessed using staining for F-actin using phalloidin and for DNA using DAPI, graph showing the change in spheroid size shown in Figure 7D; 2 mM EGTA was used as a positive control as it disrupts spheroid formation see Figure S2C for phase contrast images). Although we had employed sub IC50 concentrations, we reconfirmed whether the impairment of transition 1 and 2 was a result of dose-dependent cytotoxicity exerted by oseltamivir. When spheroids were treated similarly as in experiments of Figures 7A and 7B, and stained instead for Calcein AM (to detect viability) and propidium iodide incorporation (to detect cell death), we found that although disrupted, neither impairment nor disintegration of spheroids were accompanied by significant cell death (Figures 7E and 7F; quantification shown in Figures 7G and 7H). We also observed a disruption in spheroid formation for two other aggressive epithelial ovarian cell lines, SK-OV-3 and COV362, at a higher concentration (2 mM) of oseltamivir, which we ascertained was lower than the IC50 concentration of oseltamivir for these two cell lines 4 mM and 5 mM, respectively (Figures S3A and S4A; disruption of SK-OV-3 spheroids shown in Figures S3B and S3C; quantification in Figures S3D and S3E; disruption of COV362 spheroids shown in Figures S4B and S4C and quantification in Figures S4D and S4E). Moreover, viability assays using Calcein AM and propidium iodide showed little cytotoxicity upon oseltamivir for these two cell lines (Figures S3F and S3G for SK-OV-3 spheroids; Figures S4F and S4G for COV362 spheroids). We next asked if the spheroidogenesis-disrupting concentrations of oseltamivir have any effects on untransformed immortalized mesothelial MeT-5A cells that typify the tissue micro-environment associated with ovarian cancer metastasis. We observed extremely low levels of cell cytotoxicity exerted by 250 μM and even 2 mM oseltamivir compared with untreated controls (as assessed through Calcein AM and propidium iodide treatment (see Figure 8; and Table S5). Our results, therefore, confirm the GSMM-based predictions that oseltamivir can be a potent disruptor of ovarian cancer spheroidogenesis and its heterogeneous transitions without exerting any potently toxic side effects on the non-cancerous cells similar to those that line the coelomic cavity within which metastasis takes place.

## Discussion

Spheroidogenesis during ovarian cancer progression contributes to its recurrence, aggressiveness, and drug resistance. The molecular profiling of different stages of ovarian cancer allows a systems-level understanding of underlying mechanisms. We acquired protein expression data of three distinct morphologies, 2D monolayers, and two distinct and progressively resilient three-dimensional spheroidal states of the high-grade serous ovarian cancer line OVCAR-3. A preliminary analysis of the processed proteomic signatures between the three phenotypic states indicated that along with proteins involved in migration, drug resistance, signaling, etc., altered expression of mediators of glycolysis was associated with the phenotypic transitions. This motivated us to investigate the metabolic changes during ovarian cancer metastasis to a deeper extent. Thus, we extracted the context-specific metabolic models corresponding to each phenotypic state and evaluated the flux of reactions perturbed during the transitions. Pathway analysis from our flux evaluation showed that the significantly enriched reactions belonged to carbohydrate, amino acid, and lipid metabolism pathways. To find the core reaction modules responsible for driving the disease, we devised an approach in which perturbed reactions were clustered based on their coupled effect on the phenotypic transitions. Reactions in the top modules of both transitions included those involved in the mitochondrial and extracellular transport pathways; in contrast, the triacylglycerol (TAG) pathway was predominantly involved in the top reaction modules of the inter-spheroidal transition. The TAG pathway has been known to promote cancer growth by acquiring free fatty acids from tri-acyl glycerides to the cellular pool of fatty acids.^36^^,^^37^ In attachment-free environments, ovarian cells shift their metabolic demands on fatty acids to survive. This increased dependency on fat as an energy source can be a strategy of OVCAR-3 cells to resist anoikis, i.e., apoptosis due to cell detachment.^38^

Targeting the metabolic perturbations involved in the phenotypic transition of cancer cells can impair ovarian cancer progression toward its more metastatic and aggressive stage. Genome-level mapping of the perturbed metabolic reaction through our models provided the gene targets. Essential genes were scrupulously filtered out using the normal ovarian data from GTEx data appertaining the impact of these genes on growth rate following a knockdown. Thus, single gene knockout was performed using genes that catalyze metabolic reactions altered in morphological phenotypic transitions but are not involved in cell survival. For example, solute carrier (*SLC*) genes such as *SLC17A4, SLC17A2,* and *SLC25A1* were found to inhibit the metabolic perturbation involved in the inter-spheroidal transition significantly. Consistent with this, over-activation of *SLC25A1* has been observed in ovarian cancer, and its inhibition improved cancer cell sensitivity to platinum-based chemotherapy.^39^ Our analysis also showed that the triacylglycerol pathway (involved in lipid metabolism) was prominently involved in top disease-driving modules of transition 2, and the knockout of genes, such as *GNPAT* and *GPAM* involved in this pathway were able to block transition 2. Interestingly, it has been previously observed that *GPAM* silencing reduced cell migration and tumor xenograft development in ovarian cancer cells.^40^ Thus, the TAG pathway enhances the progression of the disease to its metastatic stage, and the genes involved in this pathway hold considerable potential to become metabolic targets. Similarly, our observations that single gene knockouts of *ALDOB, ADH1B, NEU1*, and *NT5E* block the metabolic alterations during both transitions concur with previous studies that show suppression of ovarian cancer growth upon silencing these genes.^34^^,^^41^^,^^42^ Thus, in-silico gene knockout disclosed few potential metabolic targets for drug development or repositioning.

Drug repurposing opens a feasible space for devising a single/multi-gene targeting strategy in different indications. Using the metabolic models of spheroidal morphologies, we evaluated the impact of metabolism-targeting drugs retrieved from the DrugBank database. Canagliflozin, oseltamivir, gemcitabine, and pentoxifylline target the proteins that catalyze reactions perturbed during both transitions. Among these, the drug oseltamivir (targets *NEU1*) showed larger overlap values and higher pathway perturbation. Oseltamivir is an anti-viral drug used to treat influenza A and B virus; it inhibits the neuraminidase enzyme in humans.^43^ The perturbed metabolic reactions catalyzed by neuraminidase were enriched for ECM-comprising pathways such as keratan sulfate degradation, chondroitin sulfate degradation, N-glycan degradation, and sphingolipid metabolism. Oseltamivir treatment disrupted both the single cell to moruloid and moruloid to blastuloid transitions of ovarian cancer spheroids in a dose-dependent manner in the OVCAR-3 cell line. We also observed impairment in spheroid formation and disruption of already formed spheroids in two other aggressive ovarian cancer cell lines: SK-OV-3 and COV362. No cytotoxicity was observed either in the cancer cells whose organization was disrupted or the monolayers of mesothelial, the untransformed stromal cells of the micro-environment. This validated our GSMM-based predictions that oseltamivir can be a potent disruptor of ovarian cancer spheroidogenesis. The current study thus aims to capture the crosstalk between metastatic phenotypic transitions and cognate reaction fluxes that can be extracted through context-specific metabolic modeling of protein expression data. Complementing chemotherapeutic drugs with phenotypic disruptors could help improve therapeutic outcomes in ovarian cancer management.

### Limitations of the study

Since the main focus of the present study is to develop a computational methodology using GSMM, we chose to confirm results through validation with widely used commercially available cancer cell lines. However, the promising results obtained in the *in vitro* study open new directions for researchers to take the results for further validation and evaluation through *in vitro* and *in vivo* experiments. Our current analysis looks explicitly at the phenotypic alterations associated with ovarian cancer dissemination. This could be extended in the future to the question of the metabolic changes related to carcinogenesis through a comparative analysis between untransformed fallopian cells and their transformed counterparts. We chose oseltamivir for *in vitro* validation of our model predictions because of its larger overlap values and higher pathway perturbation. However, the study offers a set of additional drugs which we predict can disrupt cancer progression through disrupting metastasis-like phenotypes. Although they have less potential, per our result, it would be worth evaluating their effect in *in vitro* and *ex vivo* model systems. Finally, our study examines and finds interventions against the metabolic flux transitions that are associated with coelomic dissemination and spheroidogenesis of ovarian cancer cells; subject to the availability of proteomic differences between untransformed and transformed ovarian surface cells, our pipeline will likely predict drugs that may impede carcinogenesis in addition to carcinomatosis. Such limitations and open questions notwithstanding, the current work provides a methodological framework for researchers to identify drug targets against ovarian cancer progression.

## STAR★Methods

### Key resources table

**Table:** 

REAGENT or RESOURCE	SOURCE	IDENTIFIER
**Chemicals, peptides, and recombinant proteins**		

Poly (2-hydroxyethyl methacrylate) (poly-HEMA)	Sigma-Aldrich	P3932
Alexa Fluor^TM^ 633-conjugated phalloidin	Thermo Fisher Scientific	A22284
DAPI (4′,6-diamidino-2-phenylindole)	Thermo Fisher Scientific	D1306
Oseltamivir phosphate	Sigma-Aldrich	SML1606
Calcein AM	Thermo Fisher Scientific	C1430
Propidium Iodide	HiMedia	TC252
Resazurin sodium salt	Sigma-Aldrich	R7017

**Deposited data**		

Proteomics data (monolayer-moruloid transition)	This work	PRIDE: PXD045194, https://www.ebi.ac.uk/pride/archive/projects/PXD045194
Proteomics data (moruloid-blastuloid transition)	Langthasa et al.^7^	PMID: 34376568
MATLAB codes	This work	GitHub: https://github.com/samrat-lab/GSMM-iScience
GTEx data	GTEx Consortium et al.,^44^ Lonsdale et al.^45^	PMID: 25954001, PMID: 23715323, https://gtexportal.org/home/datasets
Essential genes in ovarian cancer	Kanhaiya et al.^28^	PMID: 28871116
DrugBank data	Wishart et al.^19^	PMID: 16381955, http://www.drugbank.ca
GDSC data	Yang et al.^46^	PMID: 23180760, http://www.cancerRxgene.org

**Experimental models: Cell lines**		

OVCAR-3	Kind gift from Prof. Rajan Dighe, Indian Institute of Science	HTB-161
SK-OV-3	Kind gift from Prof. Rajan Dighe, Indian Institute of Science	HTB-77
MeT-5A	ATCC	CRL-9444
COV362	ECACC	07071910

**Software and algorithms**		

Recon3D model	Brunk et al.^18^	PMID: 29457794, http://bigg.ucsd.edu/models/Recon3D
Cobra Toolbox	Heirendt et al.^47^	PMID: 30787451, https://github.com/opencobra/cobratoolbox
iMAT	Zur et al.^22^	PMID: 21081510
FASTCORE	Vlassis et al.^23^	PMID: 24453953
INIT	Agren et al.^24^	PMID: 22615553
GP sampler	Herrmann et al.,^25^ Schellenberger et al.^48^	https://opencobra.github.io/cobratoolbox/
Prism	GraphPad	https://www.graphpad.com/

### Resource availability

#### Lead contact

Request for further information for resources and reagents should be directed to and will be fulfilled by the lead contact Dr. Samrat Chatterjee (samrat.chatterjee@thsti.res.in).

#### Materials availability

This study did not generate new unique reagents.

### Experimental model and study participant details

The ovarian cancer cell lines used in the study were a kind gift from Dr. Rajan Dighe (OVCAR-3 and SK-OV-3); COV362 was purchased from ECACC. The cell lines had been tested for mycoplasma contamination and also authenticated using short tandem repeat (STR) analysis.

#### Ethical approval

The studies described here were carried out in accordance with the guidelines of IISc Bioethics and Safety Committee.

### Method details

#### Mass spectrometry

The ovarian cancer cell line OVCAR-3 (obtained from ATCC, Virginia, US) was used for this study. Cells were maintained and cultured in suspension following the protocol used in our previous study.^7^ The 2D cell culture of OVCAR-3 (monolayer) in suspension aggregated into dysmorphic solid clusters, defined as moruloid. These spheroids allowed intercellular movement and penetration by new cells. Moruloid clusters then combined to form bigger clusters that matured further into blastuloid. These spheroids neither combined nor allowed penetration by new cells. Quantitative mass spectrometry was performed on three independent biological replicates of each condition. The detailed protocol used for mass spectrometric analysis is present in the study.^7^ Briefly, samples were reduced with tris(2-carboxyethyl) phosphine (TCEP), alkylated with iodoacetamide, and then digested with Trypsin. Experiments were carried out with EASY-nLC 1,000 system (Thermo Fisher Scientific) paired with a Thermo Fisher-QExactive equipped with a nanoelectrospray ion source. The spectrometric data was collected using a data-dependent top10 approach. The generated RAW files were subsequently compared to the Uniprot HUMAN reference proteome database using Proteome Discoverer (v2.2). The false discovery rate for the peptide spectrum match and the protein was set to 0.01 FDR. Statistical analysis was carried out with the help of an in-house R script. The abundance values for each run (containing all biological replicates) were filtered and imputed using a normal distribution.

#### Data pre-processing

The high throughput proteomics data contains the expression of 3122 proteins across triplicates of the distinct phenotypic morphologies monolayer, moruloid spheroids, and blastuloid spheroids. We first removed proteins absent in all nine samples of the data. Next, 2720 proteins expressing in 50% of the replicates in at least one morphological stage were filtered out. For missing value imputation, proteins expressed in at least 50% of the replicates, the missing protein expression was replaced by the mean value of the protein expression in the other two replicates. Otherwise, it was replaced by the minimum of the data. Next, the protein expression of replicates in each morphological stage was merged by taking the mean of the protein expression values of all its replicates. Gene names corresponding to the proteins present in the data were intersected with genes present in Recon3D,^18^ and 604 metabolic genes were filtered out. Finally, the data was log_10_ normalized, and the metabolic genes data was further used for building the models.

#### Fixing reaction flux bounds

We constrained the carbon-source exchanges to a maximum uptake of 10 mmol ${\text{gDW}}^{-1}$ $h^{-1}$^21^ to prevent internal reaction bounds from restricting the uptake of large polymeric carbohydrate molecules, resulting in large fluxes in the model following catabolization. Except for ${\text{CO}}_{2}$ and ${\text{HCO}}_{3}$, all model metabolites containing at least one carbon atom were considered carbon sources. Other reactions were set to the original flux bounds of the Recon3D model.

#### Mapping of expression data to reactions

In order to determine the expression data associated with each metabolic reaction, we used the GPR rules present in Recon3D. GPR rules describe the association between the reaction and the gene product catalyzing it. A function named ‘GPRparser’ of COBRA Toolbox^47^ was used to map the GPR rules to a specific format that can further be used. Then, the cell matrix containing the parsed GPR rule was used to integrate the expression data and associate it to each reaction in the model using the ‘mapExpressionToReactions’ of the COBRA Toolbox. This resulted in a reaction’s expression array, which had an expression for each reaction.

#### Integrative metabolic analysis tool (iMAT)

The iMAT implementation^22^ in COBRA Toolbox was used to extract context-specific metabolic models using expression data. The algorithm takes the following inputs.

1. model (Recon3D),
2. reaction’s expression array,
3. lower threshold (i.e., reactions with expression value below this value were considered to be non-expressed),
4. upper threshold (i.e., reactions with expression value above this value were considered to be expressed)

The algorithm aims to include the high expression reactions and remove the low expression reactions while building the model. The two threshold values used in our study for this algorithm are:

Threshold 1 (Th1): lower expression threshold: 2.4861 (minimum value of reaction’s expression array), and upper expression threshold: minimum value of top 10% of reaction’s expression array

Threshold 2 (Th2): lower expression threshold: mean(exp) − std(exp), and upper expression threshold: mean(exp) + std(exp)

where, ‘exp’ is the expression array of reactions in the model, and ‘std’ is the standard deviation.

#### FASTCORE

FASTCORE is an algorithm to build context-specific models with the advantage of fast computation and compactness of the output model.^23^ The algorithm aims to determine a set of core reactions using gene expression threshold value that are active in the output model. The algorithm also finds a minimum number of reactions to support the set of core reactions in order to make the final model consistent. The reactions with no expression data were not considered core reactions. The algorithm takes the following inputs.

1. consistent model (flux consistent subset of Recon3D),
2. core reactions (i.e., reactions with expression value above the threshold value were considered as core reactions)

The two threshold values used in our study for this algorithm are:

Threshold 1 (Th1): minimum value of top 10% of reaction’s expression array

Threshold 2 (Th2): 2.4861 (minimum value of reaction’s expression array)

#### Integrative network inference for tissues (INIT)

INIT is a model extraction algorithm,^24^ that includes and removes reactions based on the weights allotted to them, represented mathematically as:

$$\text{weight}=5\times \log_{2}\left(\frac{\text{Expression level}}{\text{Threshold}}\right)$$

where, 'Expression level' is the reaction’s expression evaluated using GPR rules.

Reactions with positive weights were considered to have high expression, whereas reactions with negative weights were considered as less expressed. A weight of −2 was given to reactions with no expression data. The algorithm ascertains the presence of highly expressed reactions by maximizing the sum of the weights of reactions. The algorithm takes the following inputs:

1. model (Recon3D),
2. weights for each reaction (must be same length as model.rxns)

The two threshold values used in our study for this algorithm are:

Threshold 1 (Th1): minimum value of top 10% of reaction’s expression array

Threshold 2 (Th2): 2.4861 (minimum value of reaction’s expression array)

iMAT has been designed to use two different threshold values (lower and upper), where the lower threshold is used to filter reactions with expressions below this value and regard them as ‘non-expressed’. While the upper threshold is used to filter reactions with expressions above this value as ‘expressed’. On the other hand, FASTCORE and INIT use a single threshold for filtering reactions based on the reaction expression. Even though iMAT uses two thresholds, it still works on involving reactions with a high expression which is the same case with the other two methods. Moreover, the threshold defining the involvement of a reaction (i.e., the upper threshold in iMAT and threshold values in FASTCORE, INIT) in all three methods is approximately the same (see Data S3 for the threshold values).

#### Evaluating steady-state flux profiles

Constraint-based metabolic models are often under-determined because they contain more number of reactions than metabolites. As a result, the solutions to such systems consist of a range of potential flux rates rather than unique flux rates for each reaction. Flux sampling is frequently used to solve genome-scale metabolic models without the use of any objective function.^25^ It generates a sequence of feasible solutions (called a chain) that satisfy the network constraints until the entire solution space is analyzed. By uniformly sampling this space, an estimated probability distribution for each reaction’s flux in the network can be obtained. To account for these feasible solutions, the uniform random sampling technique known as GP sampler is widely used and has been implemented in the Constrained Based Reconstruction and Analysis (COBRA) Toolbox.^48^ Partly based on Artificial Centering Hit-and-Run (ACHR), it samples by iteratively selecting a random direction and a random step size in that direction such that the next point also lies in the solution space. GP sampler samples the arbitrary linearly constrained space with the help of a fixed number of points (default = 2 × number of reactions in the model). The space is defined as:

Ax=b

lb≤x≤ub

The obtained solution space from the linear constrained problem is represented by:

$$E=\left\{\left(f_{1,j},f_{2,j},\ldots ,f_{n,j}\right):f_{i,j}\in R,i=1,2,\ldots ,n\right\}$$

where, *n* = number of reactions, *j* = fixed number of points.

Finally, out of the sampled solution space, the solution having a maximum correlation with the reaction’s expression array was regarded as the optimal flux solution, which is denoted by:

$$F=\left\{\left(f_{1},f_{2},\ldots ,f_{n}\right),f_{i}\in E:\;\text{corr}\left(\left(f_{i}\right),exp\right)\text{ is maximum}\right\}$$

where, i=1,2,…,n, *n* = number of reactions, exp = reaction’s expression array, corr(($f_{i}$), exp) = correlation between flux values and expression array of metabolic reactions in the queried model.

#### Evaluating metabolic functionality score

Based on biomass function, a set of 56 metabolites required for cancer growth was used to evaluate the metabolic functionalities of the extracted models. These metabolites essential for cancer cell growth include glutathione, alanine, ATP, and tyrosine. Before extracting the model, the biomass reaction was removed, and for each of these 56 metabolites, a sink reaction was included to ensure that they could be synthesized in the input model. Flux balance analysis (FBA) maximized the flux through sink reaction corresponding to each essential metabolite. If the flux value of the sink reaction was above the reaction activity threshold ($1\times 10^{-6}$), we considered the functionality as active. Each model was compared to 1000 models of the same size created by randomly eliminating reactions from the input model to determine the number of active functions. It was done to ensure the significance of the metabolic functionality score evaluated for each context-specific model. The workflow from constructing context-specific metabolic models to assessing the performance of each model, is shown in Figure S5.

#### Reaction modulation approach

Reaction modulation aims to find modules of reactions whose perturbation is associated with disease progression. Reactions perturbed during morphological transitions were considered for single reaction modulation, where each reaction was modulated individually, and the correlation between the flux states before and after modulation was evaluated against each perturbed reaction. The upper and lower bounds of the reaction modulated in the later stage model of the transition were constrained using corresponding flux values of that reaction in the initial stage model of that transition as:

if ref flux (*r*_*i*_) <0 then,
model.ub (*r*_*i*_) = 0 and model.lb (*r*_*i*_) = ref flux (*r*_*i*_)
if ref flux (*r*_*i*_) > 0 then,
model.ub (*r*_*i*_) = ref flux (*r*_*i*_) and model.lb (*r*_*i*_) = 0

where, ‘$r_{i}$’ is the reaction that is being modulated in the later stage model (i.e., ‘$r_{i}$’ belongs to the set of reactions perturbed during the considered transition), ‘model’ is the later stage model, ‘ub’ and ‘lb’ are respectively the upper and lower bounds of the reaction, ‘ref flux’ is the flux of the reaction being modulated in the initial stage model of the transition.

A separate model was built corresponding to each perturbed reaction where that particular reaction was modulated, i.e., the upper and lower bounds of that reaction were constrained using corresponding flux values of that reaction in the initial stage model of the transition. The perturbed reactions were sorted based on their correlation coefficient obtained from the single reaction modulation technique. We used models obtained from the single reaction modulation and started modulating the perturbed reactions sequentially based on their correlation values found during the single modulation technique. The correlation coefficient was evaluated on the modulation of each new reaction in the model, and the modulation was only considered significant if it increased the correlation by 0.001 (internal cut-off). Otherwise, the modulation of that particular reaction was not added to the model. Following this way, the iteration goes up to the length of the total number of perturbed reactions. The resultant model and the corresponding correlation coefficient were again put to another level of sequential reaction modulation in the same manner. This is defined as different levels of reaction modulation. So iteratively, in each level, if the sequential modulation of a reaction results in a significant increase in correlation coefficient, it was added to the model, and the model was sent to the next level until the correlation between consecutive levels becomes less than 0.01 (external cut-off).

The cut-off for reaction modulation was chosen to obtain a high correlation between profiles in a transition, with an optimal number of reactions in the modules. We evaluated various pairs of internal and external cut-offs, of which 0.001 as internal cut-off and 0.01 as external cut-off gave the highest mean correlation with a minimum mean number of reactions in the modules. The distribution of the mean correlation value and mean number of reactions in modules for different cut-offs (in both transitions) is shown in Figures S6A–S6D. For the c1 and c2 cut-off, the mean number of reactions in modules was high compared to the cut-off c3; however, the mean correlation value did not increase significantly. Conversely, for cut-off c4 and c5, the mean correlation value was decreased. This showed c3 (0.001 as the internal cut-off and 0.01 as the external cut-off) was the optimal cut-off in terms of high mean correlation with the minimum mean number of reactions in the modules. The value of all the tested cut-offs are shown in Figure S6E.

#### Filtering non-essential genes

The Genotype-Tissue Expression (GTEx) database was used to extract RNA sequence data of 166 normal ovarian samples.^44^^,^^45^ The data were quantile normalized, ensuring that the distributions were identical across samples. The conventional objective function frequently employed for biological entities is the maximization of growth rate, also known as the biomass objective function. Therefore, a model extraction method that utilizes objective function was required to find the growth rates in the normal ovarian models. Hence, we used the E-flux method to integrate data into Recon3D for generating 166 metabolic models, one for each normal ovarian sample. E-flux method uses expression data to constrain flux bounds for each reaction.^49^ The reactions catalyzed by highly expressed genes were allowed to carry higher fluxes, whereas tight constraints on the maximum flux were placed for reactions catalyzed by low expression genes. Suppose, $a_{i}$, $b_{i}$ represents minimum and maximum fluxes through the reaction $r_{i}$. Then the values of these bounds were set as follows:

For reversible reaction: $a_{i}=-b_{i}$, where, $b_{i}=f$(gene expression)
For irreversible reaction: $a_{i}=0$

To observe the knockout effect of a gene on the growth rate of normal ovarian models, the bounds of reactions catalyzed by the queried gene were set to zero. The Flux balance analysis (FBA) was then performed using Cobratoolbox’s function ‘Optimizecbmodel’ to determine the growth rate.^47^ This procedure was repeated for each gene in the normal ovarian models described above. The ratio of growth rates before and after gene deletion was then calculated for each gene in all normal ovarian models. Genes with a growth rate ratio of 1 in at least one-third of the total normal ovarian models were classified as non-essential since knocking them out did not influence the growth rate of normal ovarian models.

#### Gene knockout approach

In-silico gene knockout in metabolic models is a method of suppressing the function of a gene to assess the impact on the overall metabolic profile. To execute single gene knockout, the lower and upper flux bounds of reactions catalyzed by the queried gene were set to zero. The metabolic flux profiles were then examined and compared to the flux profiles of the transition’s reference/initial model. The metabolic model corresponding to monolayer will be the reference/initial model for transition 1, while the metabolic model corresponding to 24-h spheroids will be the reference/initial model for transition 2.

#### Approximating minimal percentage inhibition by drug targets

The aim is to determine the minimum percentage inhibition or reduction in the flux bound of reactions catalyzed by metabolic targets of the drug that can produce a maximum effect, i.e., a high correlation coefficient. We varied the percentage inhibition of the flux bounds from 10% (partial inhibition) to 100% (complete inhibition). For both transitions, the correlation coefficient between the flux values of the perturbed reactions in the drug infused metabolic model and the initial stage model was evaluated. The extent of positive correlation indicates the drug’s potential to revert the transition.

#### Cell culture and reagents

OVCAR-3 cells were maintained in RPMI-1640 (Roswell Park Memorial Institute) medium (AL162A, HiMedia) along with 10% FBS (10270, Gibco). The non-cancerous mesothelial cells, MeT-5A, were cultured in complete Medium 199 supplemented with 10% FBS, 5 μg/mL insulin, 0.5 μg/mL hydrocortisone, 2.6 ng/mL sodium selenite, 27 pg/mL β-estradiol, 10 μg/mL transferrin, 10 ng/mL hEGF, and 20 mM HEPES buffer. All the cells were maintained in a 5% ${\text{CO}}_{2}$, $37^{\circ }$ C temperature humidified incubator. For the proteomics experiments, both 2D and 3D cultures were cultivated in defined serum-free medium in order to minimize contamination in spectrometric analysis from serum proteins. Oseltamivir phosphate (SML1606, Sigma-Aldrich) was the drug used in our study. The assay medium being defined serum-free (widely used across experimental *in vitro* systems^7^^,^^50^) oseltamivir is unlikely to react with the medium. SK-OV-3 and COV362 cells were cultured in McCoy’s 5a medium (AL057A, HiMedia) with 10% Fetal Bovine Serum and DMEM (Dulbecco’s Modified Eagle Medium) medium (AL007A, HieMdia) along with 2 mm L-Glutamine, 10% FBS respectively.

#### Spheroid culture

Spheroids were cultured in 3% polyHEMA-coated (P3932, Sigma-Aldrich) dishes in basal medium supplemented with 250 ng/mL insulin (I6634, Sigma-Aldrich), 0.5 μg/mL hydrocortisone (H0888, Sigma-Aldrich), 2.6 ng/mL sodium selenite (S5261, Sigma-Aldrich), 10 μg/mL transferrin (T3309, Sigma-Aldrich), 27.3 pg/mL estradiol (E2758, Sigma-Aldrich) and 5 μg/mL prolactin (L6520, Sigma-Aldrich). These were maintained in culture for 1 to 7 days. Spheroids were visualized using the Olympus IX73 fluorescence microscope.

#### Cell staining and imaging

Spheroids were first pelleted in a sterile 15-mL tube. Cells were then fixed using 3.7% formaldehyde (24005, Thermo Fisher Scientific) at $4^{\circ }$ C for 30 min. After fixation, cells were washed and resuspended in PBS. Following this, 20 μL of spheroid suspension was dried in eight well chambered cover glass at $37^{\circ }$ C for 30 min. Permeabilization was achieved using 0.5% Triton X-100 in PBS (PBST) (MB031, HiMedia) for 2h at RT. ${\text{Alexa}\;\text{Fluor}}^{TM}$ 633-conjugated phalloidin (A22284, Thermo Fisher Scientific) was added to cells at 1:500 dilution in 0.1% PBST and incubated overnight at $4^{\circ }$ C. Cells were washed thrice with 1 × PBS for 5 min, counterstained with 1 μg/mL DAPI (D1306, Thermo Fisher Scientific).

To determine live and dead cells in the culture, cells were stained with 0.5 mg/mL Calcein AM (C1430, Invitrogen) for 15 min. Cells were washed with PBS, counterstained with Propidium Iodide (TC252, HiMedia) for 5 min, and imaged under the Olympus IX73 fluorescence microscope.

#### Resazurin assay

A cell-permeable redox indicator resazurin (R7017, Sigma-Aldrich) was used to assess the viability of ovarian cancer cells. Viable cells with active metabolism reduce resazurin to a pink fluorescent compound resorufin, and the amount of resorufin produced is proportional to the number of viable cells. The assay was performed by seeding 3000 cells per well of a 96 well plate. After 24 h, they were treated with different concentrations of oseltamivir phosphate and were incubated for 48 h. Puromycin (1 μg/mL) was used as a positive control for cell death. After the incubation period, 10 μg/μL resazurin was added to each well and incubated for 1.5 h. Fluorescence was measured using a microplate fluorometer equipped with a 560 nm excitation/590 nm emission filter set. IC50 values were calculated using a dose-response curve in Prism Graphpad.

### Quantification and statistical analysis

#### Metabolic functionality score’s significance

We assessed the significance of the metabolic functionality score evaluated for each context-specific model. We computed the metabolic functionality score of 1000 random models of the same size as the queried model under investigation. These models were generated by randomly removing reactions from the parent model Recon3D while ensuring the model’s consistency. Then, we defined the p value as follows:

$$p=\frac{\text{length}\left(\text{score}\;\left(i\right)>\text{met}\;\text{score}\right)}{\text{iter}}$$

where, ‘score’ is the metabolic functionality score for the $i^{th}$ randomly extracted model from Recon3D, ‘met score’ is the metabolic functionality score of the queried model, and 'iter' is the number of randomization (i.e., 1000 in our case).

The workflow describing the complete methodology adopted in this study and the workflow showing the steps to identify metabolic perturbations during the phenotypic transitions are provided in Supplementary (see Figures S7 and S8).

## Data Availability

• Proteomics data generated in this study has been deposited in the PRIDE database and is publicly available as of the date of publication. The accession number has been listed in the key resources table. • All MATLAB codes generated/used in this study are available on the GitHub repository: https://github.com/samrat-lab/GSMM-iScience • Any additional information required to reanalyse the data reported in this paper is available from the lead contact upon request.

## Footnotes

Supplemental information can be found online at https://doi.org/10.1016/j.isci.2023.108081. The supplementary data files (Data S1–S7) are provided in the GitHub repository: https://github.com/samrat-lab/GSMM-iScience.
