# Supplementary material for: Targeting metabolic fluxes reverts metastatic transitions in ovarian cancer
Source: iScience. 2023 Sep 28;26(11):108081. doi: 10.1016/j.isci.2023.108081 (PMC10590820; doi:10.1016/j.isci.2023.108081)
Supplement: Document S1. Figures S1–S8 and Tables S1–S5 [file mmc1.pdf]

iScience, Volume *26*

Supplemental information

Targeting metabolic fluxes reverts metastatic transitions in ovarian cancer

Garhima Arora, Mallar Banerjee, Jimpi Langthasa, Ramray Bhat, and Samrat Chatterjee

Supplementary Figures

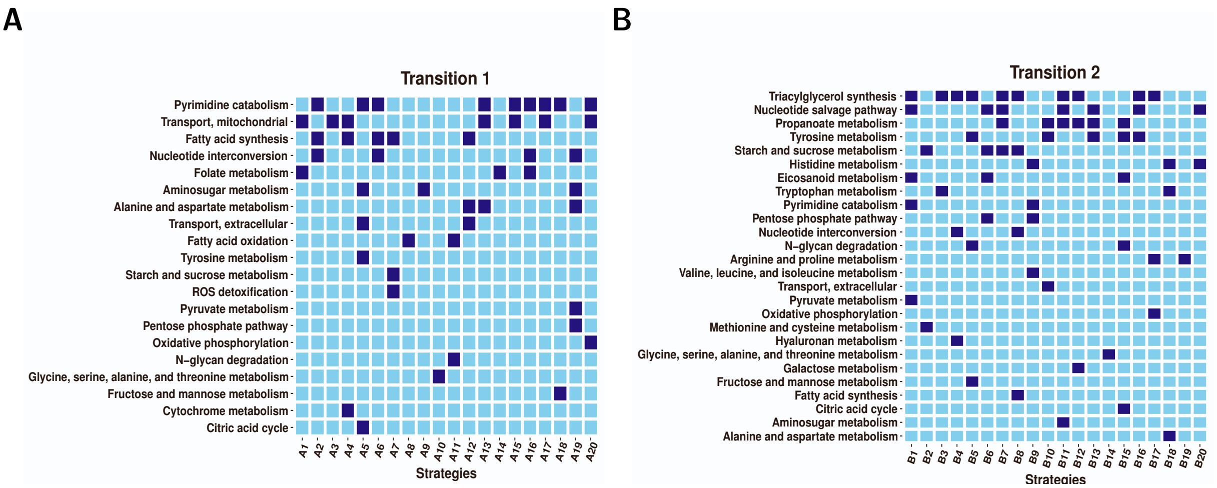

Figure S1: **: Pathway enrichment of reactions catalysed by non-essential genes (related to Figure 4):**
(A), **(B)** The figures show the enriched pathways using the reactions catalysed by non-essential genes in top 20 reaction modules of both the transitions.

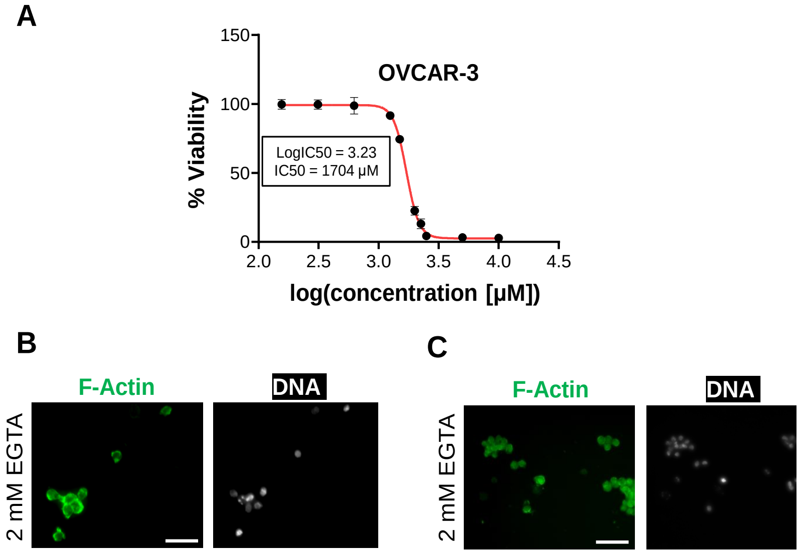

Figure S2: **Viability of OVCAR-3 cells to oseltamivir (related to Figure 7):**
(A) Graph depicting the IC50 value for oseltamivir in the ovarian cancer cell line OVCAR-3. Cells were treated with oseltamivir at progressively increasing concentrations for 48 h (n = 3).
(B), **(C)** Confocal photo-micrographs of ovarian cancer OVCAR-3 spheroids treated with 2 mM EGTA prior to (B) and post (C) spheroidogenesis (n = 3). Scale bar = 50 μm.

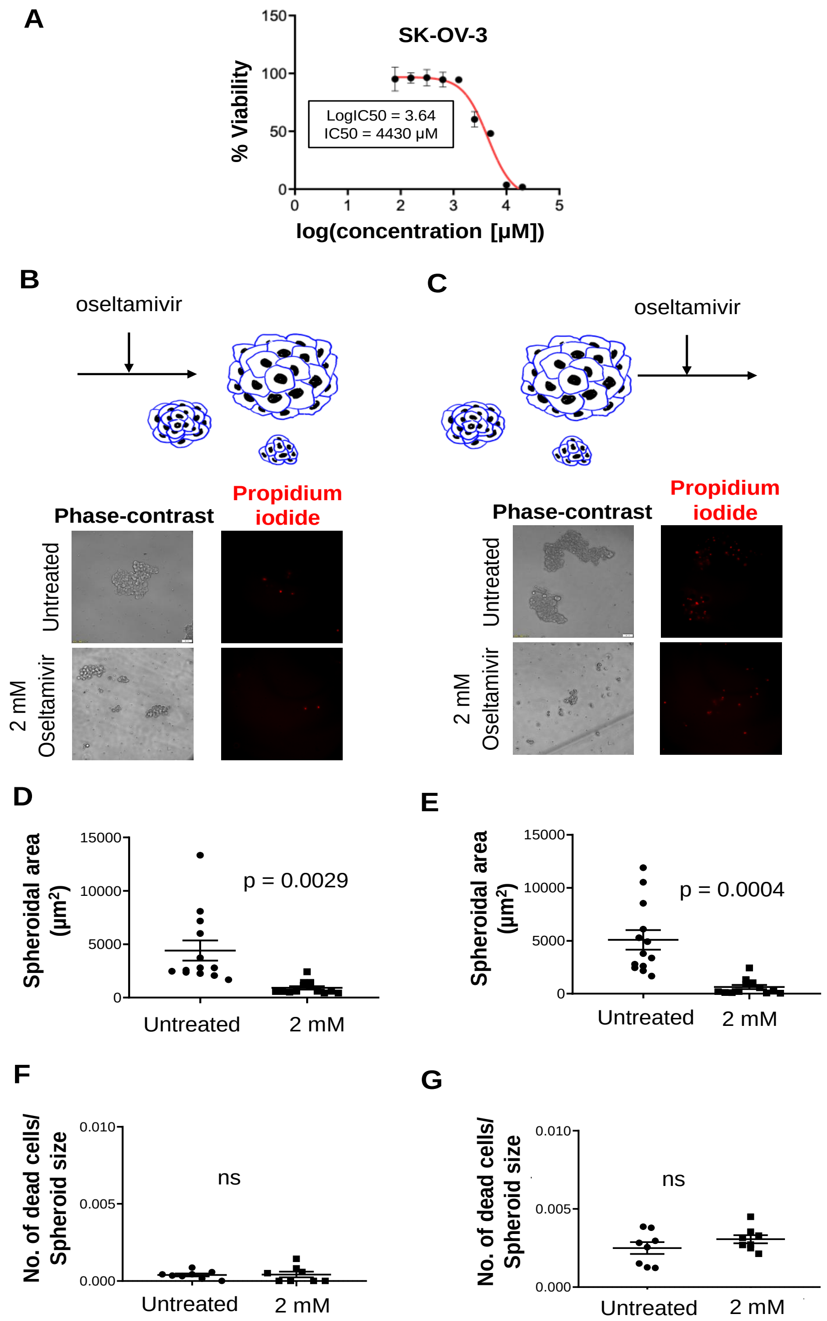

Figure S3: **Effect of oseltamivir on SK-OV-3 cell line (related to Figure 7):**
(A) Graph depicting the IC50 value for oseltamivir in ovarian cancer cell line SK-OV-3.
(B), **(C)** Fluorescence photo-micrographs of ovarian cancer SK-OV-3 cells treated with 2 mM oseltamivir prior to (B) and post (C) spheroidogenesis respectively.
(D), **(E)** Graph showing distribution of spheroidal sizes upon treatment with 2 mM oseltamivir prior to (D) and post (E) spheroidogenesis ($n = 3$, $N = 12$ spheroids).
(F), **(G)** Graph showing numbers of dead cells in a spheroid normalized to its size upon treatment with 2mM oseltamivir prior to (F) and post (G) spheroidogenesis respectively. Error bars denote mean $\pm$ SEM ($n = 3$, $N = 8$).
The unpaired Student's t test with Welch's correction was performed for statistical significance. Scale bar $= 50$ μm.

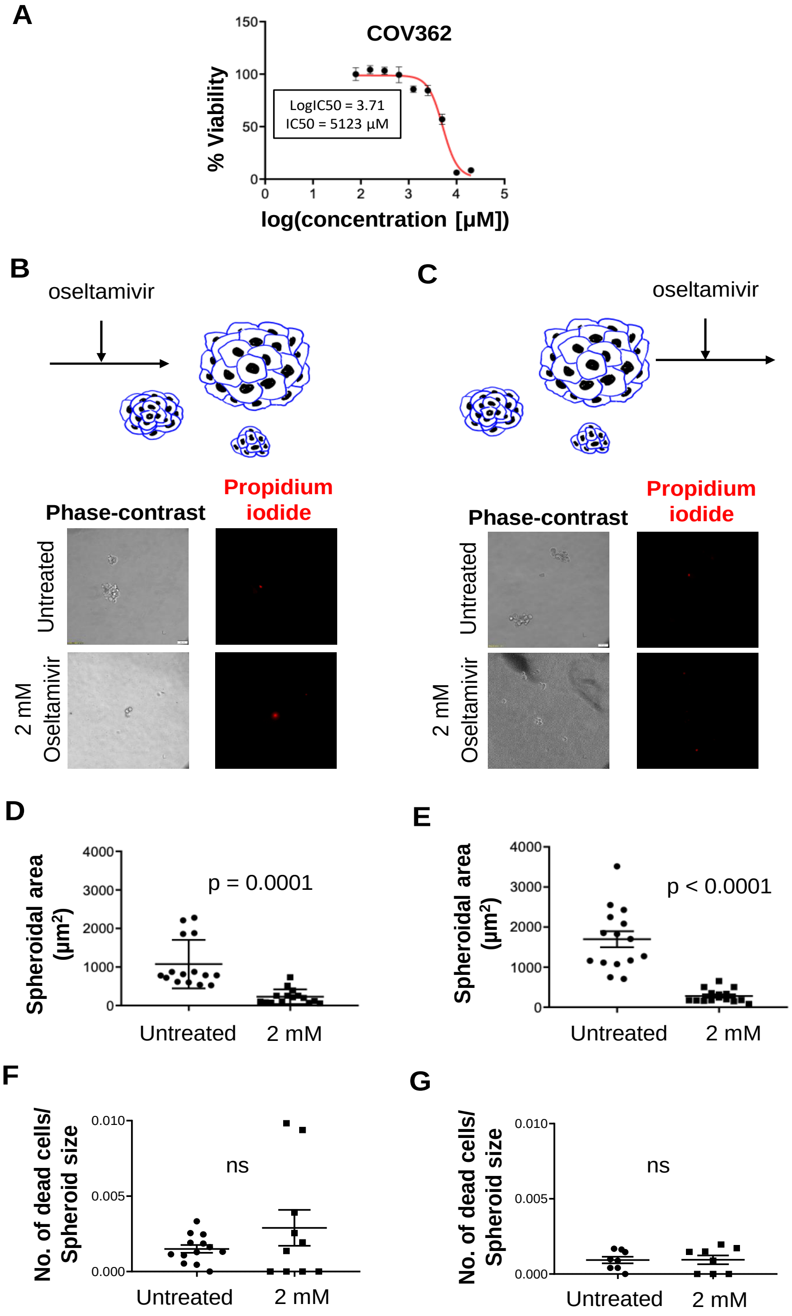

Figure S4: **Effect of oseltamivir on COV362 cell line (related to Figure 7):**
(A) Graph depicting the IC50 value for oseltamivir in ovarian cancer cell line COV362.
(B), **(C)** Fluorescence photo-micrographs of ovarian cancer COV362 cells treated with 2 mM oseltamivir prior to (B) and post (C) spheroidogenesis respectively.
(D), **(E)** Graph showing distribution of spheroidal sizes upon treatment with 2 mM oseltamivir prior to (D) and post (E) spheroidogenesis ($n = 3$, $N = 15$ spheroids).
(F), **(G)** Graph showing numbers of dead cells in a spheroid normalized to its size upon treatment with 2mM oseltamivir prior to (F) and post (G) spheroidogenesis respectively. Error bars denote mean $\pm$ SEM ($n = 3$, $N = 8$).
The unpaired Student's t test with Welch's correction was performed for statistical significance Scale bar = 50 μm.

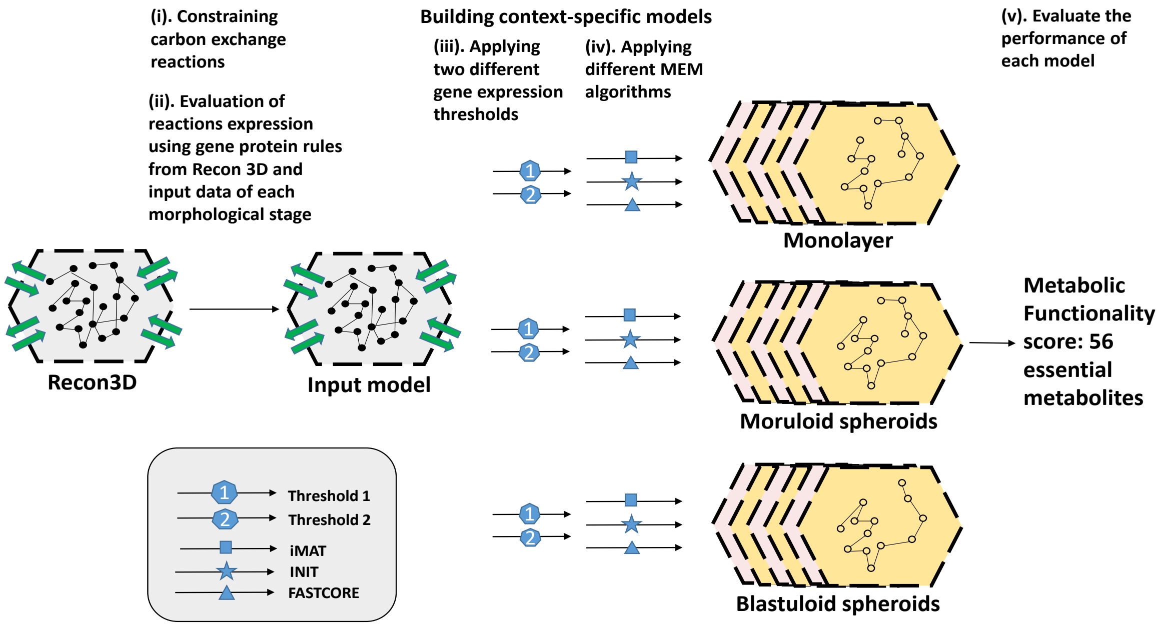

Figure S5: **Model construction workflow (related to Figure 2 and STAR Methods):** The figure describes the workflow for the construction of context-specific metabolic models by applying different MEM algorithms, gene expression thresholds and evaluating the functionality of each model.

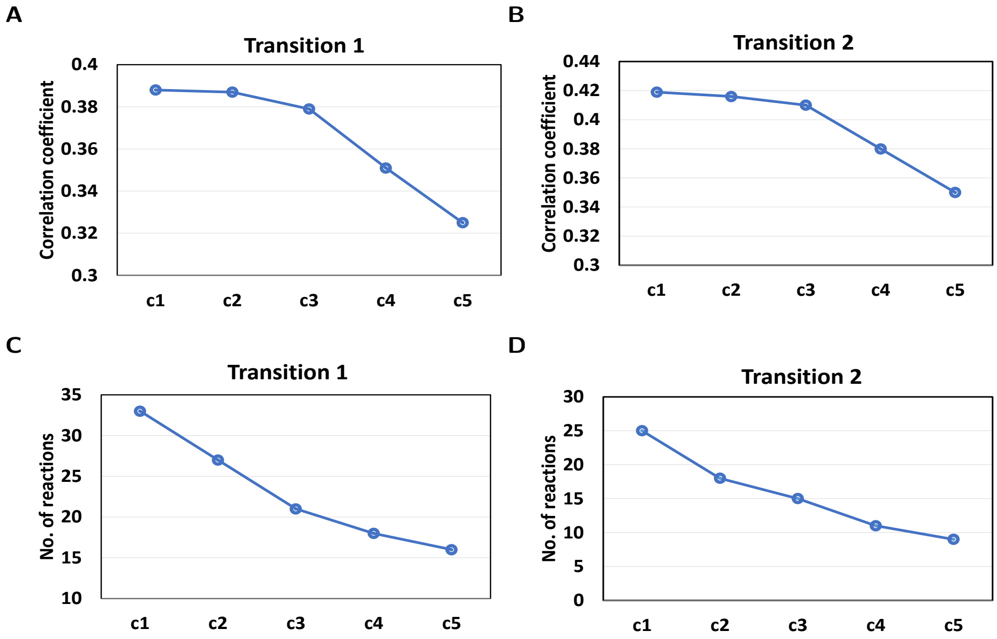

E

	c1	c2	c3	c4	c5
**Internal cut-off**	0.00025	0.0005	0.001	0.002	0.004
**External cut-off**	0.0025	0.005	0.01	0.02	0.04

Figure S6: **Cut-off selection for reaction inclusion in reaction modulation strategy (related to Figure 4 and STAR Methods):**
(A), **(B)** The figure shows the distribution of mean correlation value in modules for different sets of cut-offs in both transitions.
(C), **(D)** The figure shows the distribution of the mean number of reactions in modules for different sets of cut-offs in both transitions.
(E) The table shows the different values of internal and external cut-offs used to determine the optimal cut-off for reaction inclusion.

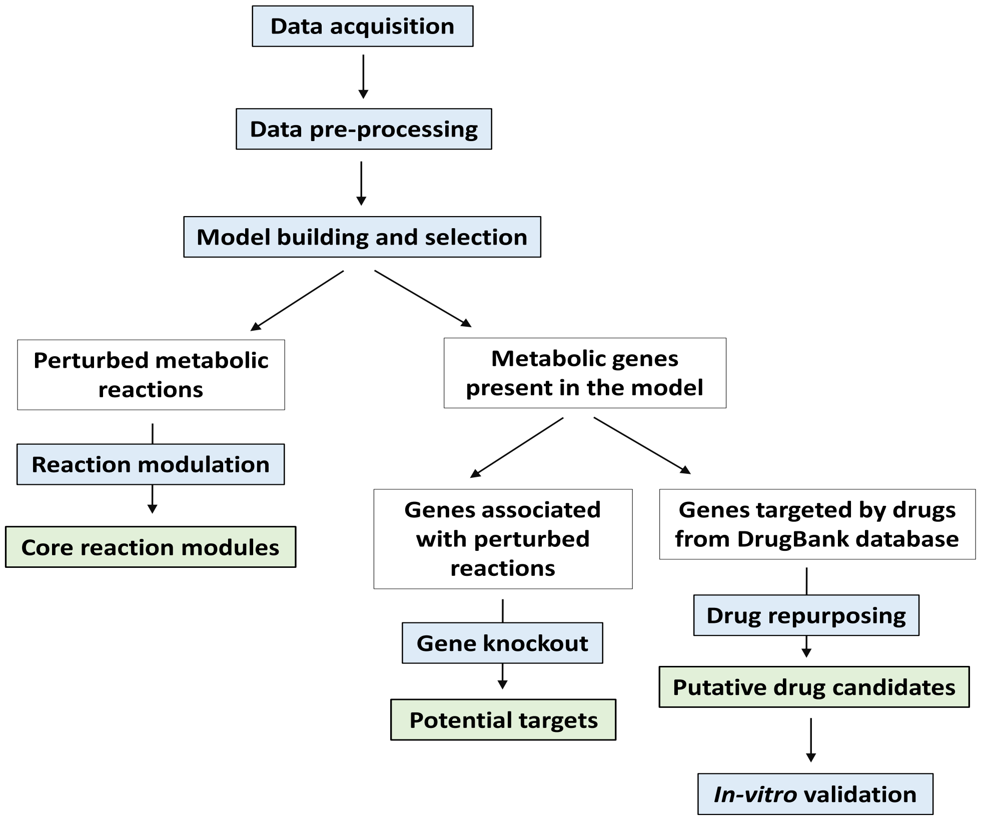

Figure S7: **Complete methodology workflow (related to Figures 1-8):**
The figure shows the complete workflow describing different methodologies adopted during the study. Genes catalyzing reactions involved in top reaction modulation strategies were among the top putative targets discovered through gene knockout analysis. Similarly, among the potential drugs that could revert the disease progression, few of them targeted genes present in the top gene knockout results.

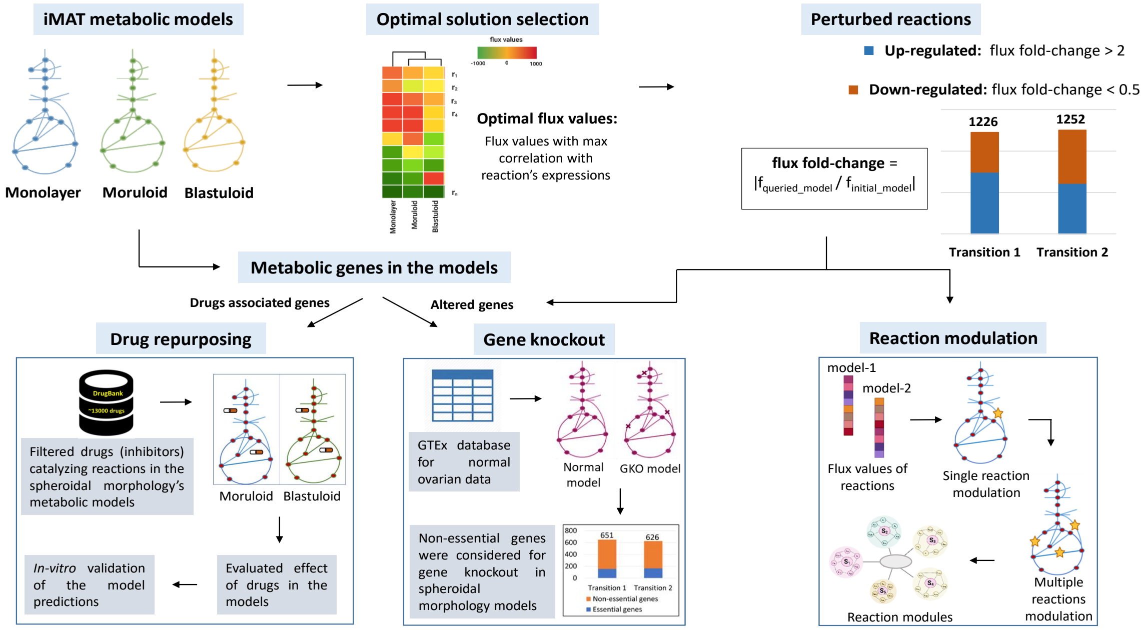

Figure S8: **Steps for finding metabolic perturbations (related to Figures 3-6 and STAR Methods):**
The flowchart showing the steps taken in order to identify metabolic perturbations, potential targets, and putative drugs that can revert the metabolic reprogramming during the phenotypic transitions.

Supplementary Tables

Table S1: **Targetability score of top 20 modulation strategies of transition 1 (related to Figure 4)**

Strategies	Targetability Score
**A1**	79
**A2**	77
**A3**	50
**A4**	63
**A5**	86
**A6**	77
**A7**	73
**A8**	71
**A9**	50
**A10**	75
**A11**	75
**A12**	90
**A13**	71
**A14**	67
**A15**	64
**A16**	74
**A17**	59
**A18**	65
**A19**	83
**A20**	91

Table S2: **Targetability score of top 20 modulation strategies of transition 2 (related to Figure 4)**

Strategies	Targetability Score
**B1**	84
**B2**	82
**B3**	61
**B4**	79
**B5**	68
**B6**	79
**B7**	65
**B8**	83
**B9**	68
**B10**	83
**B11**	71
**B12**	80
**B13**	69
**B14**	64
**B15**	73
**B16**	73
**B17**	73
**B18**	68
**B19**	76
**B20**	76

Table S3: **In-silico validation using GDSC sensitivity score (transition 1) (related to Figure 6)**

Drugs	Drug sensitivity score	Correlation after drug addition
**Afatinib**	-0.019774	0.1963
**Bosutinib**	0.98623	0.0309
**Cetuximab**	0.36838	0.0276
**Crizotinib**	1.07	0.0276
**Cytarabine**	0.70312	0.0841
**Dabrafenib**	0.6468	0.0276
**Dasatinib**	-0.50914	0.1963
**Gemcitabine**	0.92802	0.2155
**Ibrutinib**	0.34828	0.0276
**Lapatinib**	1.0507	0.1963
**Leflunomide**	0.26131	0.2508
**Pazopanib**	-0.29808	0.2101
**Pemetrexed**	0.44992	0.0903
**Ponatinib**	0.42051	0.0276
**Ruxolitinib**	0.37862	0.0276
**Sorafenib**	-0.17518	0.1931

Table S4: **In-silico validation using GDSC sensitivity score (transition 2) (related to Figure 6)**

Drugs	Drug sensitivity score	Correlation after drug addition
**Afatinib**	-0.019774	0.2051
**Bosutinib**	0.98623	0.0389
**Cetuximab**	0.36838	0.1887
**Crizotinib**	1.07	0.0389
**Cytarabine**	0.70312	0.0352
**Dabrafenib**	0.6468	0.1887
**Dasatinib**	-0.50914	0.2033
**Gemcitabine**	0.92802	0.1851
**Ibrutinib**	0.34828	0.1887
**Lapatinib**	1.0507	0.2051
**Leflunomide**	0.26131	0.1372
**Pazopanib**	-0.29808	0.1993
**Pemetrexed**	0.44992	0.1537
**Ponatinib**	0.42051	0.1887
**Ruxolitinib**	0.37862	0.1887
**Sorafenib**	-0.17518	0.2088

Table S5: **Cell viability of untransformed mesothelial MeT-5A cells after oseltamivir treatment (related to Figure 8)**

	% Viability (mean $\pm$ SEM)	% Dead cells (mean $\pm$ SEM)
**Untreated**	98.748 $\pm$ 0.082	1.252 $\pm$ 0.082
**250 μM Oseltamivir**	98.72 $\pm$ 0.191	1.278 $\pm$ 0.191
**2000 μM Oseltamivir**	96.729 $\pm$ 0.346	3.217 $\pm$ 0.346
